# Glycyrrhizic Acid Class Compounds in Immune Checkpoint Inhibitor Therapy: Stereochemistry, Mechanistic Hypotheses, and Protection Against Immune-Related Organ Injury

**DOI:** 10.3390/ijms27219987

**Published:** 2026-11-07

**Authors:** Jie Li, Baojian Li, Ying Gao, Boshen Zhao, Jasur Kasim, Nanqi Shao, Zheng Gao, Zhan Liu, Zhongxing Song, Yinglai Yang, Jianguang Li

**Affiliations:** 1 Science and Innovation Center of Xinjiang Second Medical College, Karamay 834000, China; lijie@xjsmc.edu.cn (J.L.); libaojian@xjsmc.edu.cn (B.L.); gaoying@xjsmc.edu.cn (Y.G.); zhaoboshen_0728@163.com (B.Z.); jasur2025@163.com (J.K.); shaonanqi2024@126.com (N.S.); 18963362670@163.com (Z.G.); liuzhan365@163.com (Z.L.); szx74816@sina.com (Z.S.); 2 School of Pharmacy, Xinjiang Second Medical College, Karamay 834000, China

**Keywords:** licorice, glycyrrhizic acid, magnesium isoglycyrrhizinate, immune checkpoint inhibitor, immune-related adverse events, HMGB1

## Abstract

Glycyrrhizic acid class compounds are used empirically for checkpoint inhibitor-induced liver injury (ChILI), but their effects on antitumour efficacy remain uncertain. This narrative review examines isomer-specific pharmacology, clinical evidence and the candidate mechanisms proposed for this class, of which the high-mobility group box 1 (HMGB1) axis is the most developed. In a propensity-score-matched cohort of 229 patients, magnesium isoglycyrrhizinate (MgIG) plus corticosteroids was associated with higher day-10 alanine aminotransferase normalization than corticosteroids alone (30.7% versus 17.4%). A single-arm study of 67 patients reported study-defined liver injury in 9.0% during prophylaxis. Neither study reported antitumour efficacy or established causal clinical benefit. Modulating extracellular HMGB1 might reduce organ inflammation while altering tumor immunosuppression. However, neither direct HMGB1 engagement by MgIG nor a causal contribution of HMGB1 to ChILI was established in the identified literature. HMGB1 can also support antitumour immune priming; its net effect depends on redox state, tissue and timing. The corresponding 18α aglycone shows no measurable inhibition of 11β-hydroxysteroid dehydrogenase type 2 up to 20 µM in vitro, which does not establish clinical safety. Controlled trials should assess liver injury, corticosteroid exposure, antitumour outcomes and electrolyte safety together; routine prophylaxis is not supported.

## 1. Introduction

Immune checkpoint inhibitors (ICIs) have reshaped the treatment of advanced malignancy, and with them has come a distinct pattern of immune-mediated toxicity. Liver injury of this kind—checkpoint inhibitor-induced liver injury (ChILI)—is classified as an indirect drug-induced injury, and among patients given ICIs its reported incidence runs from 2% to 25% [1]. Injury of grade 2 or higher can require withholding of ICIs and, in some patients, permanent discontinuation, removing a potentially life-prolonging therapy from a responding patient.

Corticosteroids are the recommended first-line treatment, but their role is not settled. In a multicentre retrospective cohort of 20,163 veterans receiving ICIs, steroids given for immune-related adverse events (irAEs) were associated with better survival than steroids given for other reasons or no steroids at all—but among those treated for irAEs, early exposure, within two months of starting the ICI, was associated with a reduced relative survival benefit compared with later exposure, whether or not the ICI was continued afterward [2]. Escalating the dose, for its part, has not consistently produced better outcomes as Zhi et al. record in reviewing earlier work [1]. A steroid-sparing agent that resolves hepatotoxicity without blunting antitumour immunity would therefore meet a real clinical need.

The glycyrrhizic acid class is an unexpected candidate. Its most widely used member, magnesium isoglycyrrhizinate (MgIG), holds a Chinese approval for acute drug-induced liver injury [3], and a review cited here records that the 2023 national guideline on that condition names it as the only approved agent for the indication [4]; in ChILI the class is used empirically by clinicians [1]. In ICI-treated populations, MgIG has been studied as treatment, in a multicentre propensity-score-matched analysis [1], and as prevention, in a prospective single-arm study in which study-defined liver injury arose in 9.0% (6/67, 95% confidence interval (CI) 3.4–18.5%) [5]. The pharmacological bridge between this class and checkpoint inhibitor therapy while also asking whether protecting the liver preserves or compromises checkpoint blockade.

Eight reviews have surveyed licorice constituents in oncology [6,7,8,9,10,11,12,13], and a more recent one, of glycyrrhizin preparations in drug-induced liver injury rather than in oncology, states that MgIG-based regimens are used against liver injury induced by immune checkpoint inhibitors [4]. None of the reviews identified by this search assembles the pharmacological bridge between this class and checkpoint inhibitor therapy, or asks whether protecting the liver preserves or compromises checkpoint blockade; nor does the review that makes the connection include the peer-reviewed checkpoint-inhibitor datasets assembled in Section 7. That question is the gap—a finding of the search specified in Section 2, not a claim about all literature.

This review addresses that question: the antitumour-immunity literature built on these compounds (Section 5) and their use as delivery platforms (Section 6); the evidence for protection against immune-mediated organ injury (Section 7); the mechanism that may unify antitumour immunity and immune-mediated organ injury (Section 4) and what the assembled evidence makes of it (Section 8); the resulting safety question (Section 9); and the studies that would test it (Section 12). Section 5.2 sets apart constituents of *Glycyrrhiza* that do not belong to this class, because their pharmacology cannot be pooled with it.

The class is pharmacologically heterogeneous: the stereochemistry of the aglycone determines which enzyme it inhibits, with consequences for both efficacy and toxicity (Section 3.1), and treating “glycyrrhizic acid” as a single entity without stating the isomer or the preparation limits extrapolation across isomers and formulations. The mechanistic framework proposed in Section 4 is a hypothesis derived by synthesis, and Section 8 sets out the predictions that would falsify it; statements that a study or measurement is missing are bounded by the search described in Section 2.

## 2. Literature Search and Evidence Appraisal

### 2.1. Search Strategy

This is a narrative review, not a systematic review. It follows no PRISMA (Preferred Reporting Items for Systematic Reviews and Meta-Analyses) protocol, and we make no claim to the completeness such a protocol would provide. The search is set out explicitly all the same, because a statement that something is missing is worth only as much as the search behind it.

We searched PubMed/MEDLINE, Europe PMC, PubMed Central, Crossref, OpenAlex and the Directory of Open Access Journals (DOAJ) for records from database inception to 24 September 2026, with no language restriction. The concept-block searches were run in PubMed/MEDLINE; the other sources were queried and used to identify records, to obtain full texts and to verify bibliographic and open-access status. Chinese-language literature was reached through the same international indexes and, where a record was indexed only in a Chinese database, through the corresponding SinoMed, China National Knowledge Infrastructure (CNKI) or journal-site record. Full text was reviewed wherever it could be obtained, and evidence resting on an abstract alone is labeled as such in the text and in the tables. The sources are at https://pubmed.ncbi.nlm.nih.gov (PubMed/MEDLINE), https://europepmc.org (Europe PMC), https://pmc.ncbi.nlm.nih.gov (PubMed Central), https://www.crossref.org (Crossref), https://openalex.org (OpenAlex) and https://doaj.org (DOAJ), all accessed on 24 September 2026.

Records were retrieved with three concept blocks—substance, clinical context, and mechanism and organ—combined in several ways rather than only as a single intersection. Naming agents individually in the clinical-context block broadens the clinical retrieval: in the largest clinical dataset on this question, the most common causative agents were camrelizumab, sintilimab and tislelizumab [1]. The complete term list for each block, and the Boolean string of every PubMed query, are given in Section S1 of the Supplementary Material.

In PubMed/MEDLINE the substance and clinical-context blocks intersected in 23 records at the cutoff, and adding liver-injury or immune-related-adverse-event terms returned one—the multicentre case–control study of Zhi 2025 [1]; the other 22 are mechanism studies, nanoplatform designs and reviews of adjacent constituents. Those counts are bounded by four limits: they are PubMed/MEDLINE counts, and the other sources were not counted; the searches covered titles and abstracts only; the Chinese-language literature could not be searched with the same query syntax, so that part is not reproducible in the same way; and screening was not performed in duplicate. This review therefore makes no completeness claim about randomized comparisons or safety series published only in Chinese.

Substance-only and context-only searches were run deliberately: requiring both blocks in one query would have excluded precisely the papers this review exists to connect. We then snowballed—screening the reference lists and citing articles of the index papers—and repeated targeted searches whenever a statement turned on whether a particular study or measurement exists.

### 2.2. Inclusion Criteria

A study was included if it reported a clinical or experimental outcome—efficacy, safety, pharmacokinetics, or a mechanistic readout with a defined endpoint—for either a glycyrrhizic acid class compound or an adjacent *Glycyrrhiza* constituent. Several decisions shape the tables and are stated here explicitly:

- **Adjacent constituents.** Terpenoids, flavonoids and polysaccharides of *Glycyrrhiza* that are not members of the glycyrrhizic acid class are admitted only as adjacent evidence. They are segregated in Section 5.2 and in panel E of Table S2, and are never counted as evidence for this class. They are included at all because the clinical and preclinical literature routinely treats “licorice” as a single agent.
- **Delivery systems** (Section 6). A nano-delivery study was included only where a glycyrrhizic acid class compound is a structural, targeting or functional component of the system and the study reports an immune or antitumour endpoint. Studies in which the compound is a passive excipient, or whose only endpoints are physicochemical, were excluded.
- **Abstract-level sources**. Four are cited only from their abstracts [14,15,16,17]: three are conference proceedings with no pagination, marked [conference abstract] in the reference list, and one is an indexed journal item marked [abstract only] [16] because no full text was obtained and the record type could not be established. Reading only an abstract does not establish that the article is an abstract. Two report a preclinical measurement for which no peer-reviewed report could be retrieved; the other two are versions of work that later appeared in full—[15] as [18], and [17] as [19]—and each is cited only where it is read with its full report. They are excluded from every quantitative claim and from every recommendation. Two further references entered this review after the search cut-off, cited for general properties of high-mobility group box 1 (HMGB1) signaling and for no numerical value (Section 4.3): [20] was read from the open-access full text in Europe PMC, and [21] from the article text obtained through an interlibrary document service.
- **Primary and secondary sources**. Primary studies form the basis of the mechanistic and clinical evidence summaries. Reviews and meta-analyses are used for background, for aggregated safety information and for citation tracking, and where one is discussed its evidence type is identified.
- **Exclusions**. Phytochemical and analytical reports without a biological endpoint; in vitro cytotoxicity screens without an immune or organ-injury readout; narrative reviews without primary data, which were used for background and citation tracking as described above; and commercial or promotional material, with one deliberate exception—the manufacturer’s product information and the national reference standard for magnesium isoglycyrrhizinate are used in Section 3.3 as the regulatory source of an approved chemical identity, and are labeled as regulatory rather than experimental sources.

### 2.3. Statements of Absence

“Not identified” is not “does not exist”. Negative statements appear in three grades, and they are not interchangeable:

- **Not identified by our search**—the only negative claim a search can support. Where the text says that no study was identified, that is what is meant, on the strength of the search described above and its last-search date.
- **Not reported in an identified study**—for example, progression-free and overall survival were not reported by either of the two clinical datasets that defined liver-injury endpoints. This is a claim about those publications and is verifiable directly from them.
- **Testable but not yet tested**—the measurement has not been made, and the design or material that would permit it is in principle available. The serum-HMGB1 analysis proposed in Section 12 is of this kind.

Statements of the first kind are date-stamped: a single paper published before 24 September 2026 that reports a measurement we describe as missing would falsify such a statement, so that a reader can check it.

### 2.4. How the Evidence Is Described

This review applies no summary grade: a single scale would rank studies that answer different questions, and regulatory documentation of a product’s chemical identity documents the product rather than an effect. The appraisal in Section 10 sets out, for each question, the direct evidence that exists, its principal limitations and the conclusions it does not support—the design and comparator, the sample size, the match between population, outcome and question, the precision of the estimate and the main sources of bias—and says so where no direct evidence exists. Section S2 of the Supplementary Material explains how each evidence label was assigned.

The two datasets that define liver-injury endpoints are a retrospective propensity-score-matched case–control study and a single-arm study, and the prospective comparative data concern populations other than ICI-treated patients. The licensed product’s chemical identity is documented by regulatory sources [3,22] and is reported beside the appraisal in Section 10 as a product fact rather than assessed as evidence about an effect.

### 2.5. Limitations

This is a narrative review, not a systematic one: it follows no PRISMA protocol and makes no claim to completeness. The counts reported above are PubMed/MEDLINE counts; the other sources were not counted. Searches covered titles and abstracts, screening was not performed in duplicate, and the Chinese-language literature could not be queried with the same syntax, so that part is not reproducible in the same way. Four references are read from abstracts alone and are labeled as such; they support no quantitative claim and no recommendation.

## 3. The Glycyrrhizic Acid Class Is Not Pharmacologically Homogeneous

### 3.1. Stereochemistry and Implications for Safety

Licorice accumulates glycyrrhizin, whose aglycone is the 18β form of glycyrrhetinic acid (18β-GA) [23]. This natural isomer inhibits both isozymes of 11β-hydroxysteroid dehydrogenase (11β-HSD1 and 11β-HSD2), preferentially the isozyme whose inhibition produces mineralocorticoid excess—a risk signal for this isomer rather than a property that predicts how well it protects the liver. Transfected HEK-293 lysates gave the 18β aglycone an IC_50_ of 779 nM (95% CI 632–960 nM) at human 11β-HSD1 and 257 nM (95% CI 186–354 nM) at 11β-HSD2—approximately threefold greater potency at the second enzyme, which the authors report as a statistically significant difference [23].

That preference is the molecular basis of the best-known toxicity of licorice. 11β-HSD2 inactivates cortisol to cortisone in the kidney; when it is inhibited, cortisol escapes inactivation and activates the mineralocorticoid receptor, producing the hypernatremia, hypokalemia and hypertension of mineralocorticoid excess [23]—the mechanism behind the pseudoaldosteronism of licorice excess, whose clinical reports are cited below. Case reports of the syndrome continue to appear [24,25,26,27], and insulin-treated diabetes has long been recognized as a susceptibility factor [28].

The corresponding 18α aglycone behaves differently. Classen-Houben and colleagues assayed the two aglycones, not the glycoside: purified 18α-glycyrrhetinic acid (18α-GA) and 18β-GA against human 11β-HSD1 and 11β-HSD2 in transfected HEK-293 lysates. That assay places the 18α aglycone at an IC_50_ of 2214 nM (95% CI 1654–2963 nM) against 11β-HSD1, with no measurable inhibition of 11β-HSD2 even at 20 µM [23]—the property the authors describe as “the favorable property of not inhibiting 11β-HSD2”. Selectivity costs roughly threefold lower potency against HSD1—a favorable trade if the aim is to modulate HSD1 without incurring mineralocorticoid toxicity.

The assay used the aglycone, not the preparation. The preparation is a glycoside—the magnesium salt of 18α-glycyrrhizinate—and 18α-GA is the aglycone released from it by hydrolysis, measurable in plasma after intravenous administration in rats [29], as the aglycone of glycyrrhizin is after oral dosing [30]. The HSD2 measurement therefore applies to the corresponding 18α aglycone and not to the intact MgIG molecule, whose own HSD2 profile no source appraised here measures: it is an in vitro difference in enzyme inhibition between aglycones, not a clinical mineralocorticoid-risk estimate for the preparation. The clinical layer is separate and weaker: 18β glycyrrhizin preparations cause pseudoaldosteronism in patients [28], MgIG’s laboratory safety profile in the one ICI cohort was comparable to steroid monotherapy [1], and MgIG’s own record is not uniformly reassuring [5,31]. No study compares isomer-defined preparations on mineralocorticoid endpoints, so a lower risk for MgIG remains a hypothesis from the aglycone assay (Section 11.1).

The same isomerization underlies the licensed preparation. MgIG is produced by alkali-catalyzed isomerization of 18β-glycyrrhizic acid followed by salification [1]; and the chemistry used to access 18α-GA experimentally—treatment of 18β-GA with KOH in diethylene glycol at 220 °C [23]—is an analogous transformation on the aglycone. The descriptions of the licensed product’s configuration are set out in Section 3.3.

The practical implication is threefold. First, mechanistic findings obtained with glycyrrhizin or 18β-GA cannot be transferred to MgIG without qualification, because the two differ in which HSD isozyme they inhibit. Second, the HSD2-sparing profile of the 18α aglycone rests on the aglycone assay and has no clinical counterpart: the ICI cohort’s reported safety panel was complete blood count and renal function, and reported no potassium, blood pressure or aldosterone outcomes, so it can be read neither for nor against HSD2 sparing in vivo. Third, consumers taking unstandardized licorice products, in which the 18β isomer predominates, remain exposed to the mineralocorticoid risk that the 18α preparations were expected largely to avoid, an expectation the clinical data have not established (Section 11.1).

### 3.2. Alternative Mechanistic Arms: Bile-Acid Receptors and 11β-HSD1

The hepatoprotective action of this class is not exhausted by anti-inflammatory signaling. In concanavalin A (ConA)-induced immune liver injury, MgIG attenuated transaminase elevation and hepatocellular necrosis while upregulating hepatic farnesoid X receptor (FXR) and pregnane X receptor (PXR) expression, read as enhanced bile-acid metabolism [14]; the same model also showed lower TNF-α and IFN-γ with reduced oxidative and inflammatory markers [18].

This is relevant in ChILI, where hepatotoxicity often has a cholestatic or mixed phenotype that anti-inflammatory signaling alone would not address.

A second candidate is better supported, although no study appraised here tests it in ChILI. MgIG binds 11β-HSD1 (HSD11B1) directly at lysine 187 (Lys187), corroborated by molecular dynamics, and alleviates alcohol-associated liver disease through the HSD11B1–SREBP2–IDI1 axis [32]; separately, the 18α aglycone inhibits the same isozyme in vitro [23] (Section 3.1).

The FXR/PXR arm rests on the weakest evidence here: the receptor measurements come from a 2008 conference abstract [14], are not reproduced in the peer-reviewed report of the same model [18], and a second conference abstract reports mitogen-activated protein kinase (MAPK) involvement [17]. No quantitative claim or recommendation rests on it.

Neither arm is subordinate to the HMGB1 axis of Section 4: they are candidate mechanisms in parallel, and no study identified here separates their contributions.

### 3.3. Pharmacokinetics, Metabolism and Formulation

Orally administered glycyrrhizin is poorly absorbed, which is why the clinical literature is dominated by an intravenous preparation: intestinal bacteria hydrolyze it to GA, the species absorbed and the principal active metabolite, so exposure depends on microbiota composition as well as on the dose swallowed. Antibiotic perturbation altered glycyrrhizic acid bioavailability in rats [33], *Lactobacillus murinus* colonization improved its absorption [34], and in a Kampo medicine plasma GA varied with microbiota composition and fell with some antibiotics but not others [35]. In an oncology population, this variability argues against oral licorice products in place of a defined preparation.

In rats, oral dosing with the mono-glucuronide metabolite 18β-glycyrrhetic acid 3-O-mono-β-D-glucuronide itself yields far higher plasma concentrations of that metabolite than oral dosing with glycyrrhizin does: the two peaks are concentrations of the same analyte, and differ only in which substance was swallowed—2377 ng/mL at 5 min after the metabolite, against 346 ng/mL at 2 h after glycyrrhizin [30]. The source’s area under the concentration–time curve (AUC) values are inconsistent with its own maximum plasma concentration (C_max_) values and are not interpreted quantitatively anywhere in this review (Section S3); the peak concentrations carry the comparison. Dose, compound form and microbial conversion therefore act together on exposure after oral administration.

MgIG is given intravenously, bypassing these steps [1,5], and binds human serum albumin at a single site (site I) [36]. Single infusions of 100–300 mg in healthy volunteers gave mean peak plasma concentrations of 28.79–99.28 mg/L with a terminal half-life of about 19–31 h [37]; those are total plasma concentrations, not free concentrations at the site of action, and they are not comparable with affinities measured in vitro for a different entity (Section 4.1). A meta-analysis of ten studies and 1522 patients with chronic liver disease found greater reductions of alanine aminotransferase (ALT), aspartate aminotransferase (AST) and bilirubin at 200 than at 100 mg/day, the closest thing to dose-finding for the class, though not in an oncology population [31]. Hepatic targeting can be conferred deliberately: liposomes modified with a GA derivative were directed to the liver through the binding and uptake mechanism reported for glycyrrhetinic acid derivatives [38].

Glycyrrhizic acid is amphiphilic and self-assembles into nanofibrils above a critical aggregation concentration [39], and the class is not pharmacokinetically inert toward co-administered drugs: glycyrrhizin with high-dose methotrexate raised methotrexate concentration and half-life and worsened hepatic injury in rats, whereas separating the doses avoided the interaction [40], and in a separate rat study GA increased warfarin exposure [41]. In humans the question has been examined once, in a pilot study of 18 patients with epithelial ovarian cancer (9 per arm) receiving paclitaxel plus cisplatin with intravenous MgIG 100 mg or vehicle: paclitaxel’s terminal half-life fell from 11.0 ± 2.2 to 7.7 ± 1.7 h (*p* = 0.002) and its mean residence time from 5.6 ± 1.0 to 4.0 ± 0.3 h (*p* = 0.001), while AUC_0–48_ h fell from 9734 ± 3513 to 7214 ± 2167 ng·h/mL without reaching significance (*p* = 0.103) [42]. A shortened half-life with an exposure change that does not reach significance is a pharmacokinetic signal rather than evidence that antitumour efficacy falls.

Finally, the pharmacological literature is not consistent about which isomer the licensed preparation contains. Four descriptions support the 18α assignment—the original pharmacological characterization [18], two reports naming the preparation as the 18α salt [43,44], and an optimized synthesis whose product and key intermediates were confirmed by ^1^H NMR, ^13^C NMR and HRMS [45]—and two further peer-reviewed reports describe the preparation itself as an 18α salt [19,46]. Against these, one physicochemical characterization describes it as the 18β salt [36], which conflicts with the approved identity of the licensed product: the manufacturer product information specifies the (18α,20β) configuration [3], and the national reference standard is filed under the same name and molecular formula [22]. We therefore treat the 18α assignment as established by the regulatory identity of the licensed product, and the 18β description as a misassignment.

The wider point is that if a primary physicochemical report can misstate the configuration of a licensed drug, isomer identity is a specification journals should state explicitly, as Section 11.5 recommends for product labeling.

## 4. The HMGB1 Axis as One Candidate Mechanism

### 4.1. HMGB1 as a Damage-Associated Molecular Pattern, and the Glycyrrhizic Acid Class as Its Inhibitor

When cells are injured or activated, they release HMGB1, which outside the cell behaves as a damage-associated molecular pattern, signaling through Toll-like receptors (TLRs) and through the receptor for advanced glycation end products (RAGE) [47,48,49]. Glycyrrhizin binds HMGB1 directly and inhibits its cytokine activities [50]; it is the best-characterized small-molecule HMGB1 inhibitor, but its affinity is weak. NMR gives dissociation constants of 103 ± 25 µM and 87 ± 35 µM for the two isolated HMG boxes, fluorescence gives 170 ± 3 µM and 140 ± 3 µM for the same boxes and 156 ± 3 µM for the full-length protein [50], and structure-based derivatives have improved on this only to 12.5 µM; the same series measures the aglycone by surface plasmon resonance at *K_d_* 73.3 µM, against the ≈150 µM value it quotes for the glycoside, without stating the aglycone’s isomer [51]. An affinity constant settles nothing on its own: those values belong to the glycyrrhizin glycoside or to an aglycone whose isomer is unstated, while clinical exposure is of the licensed glycoside MgIG. What matters is whether the free concentration of the entity whose *K_d_* was measured reaches its functionally effective concentration at the site of action, and two facts constrain that comparison without settling it: MgIG binds human serum albumin at a single site [36], so its free concentration is below its total, and it is given intravenously at 200 mg/day [1], which is a dose rather than a concentration. What remains unknown is the free concentration of the active entity, its exposure in the target tissue, and whether it engages HMGB1 functionally in patients; until those are known, the affinity measurements bound what the class could do rather than what it does. In experimental sepsis, lipopolysaccharide reached the cytosol by an HMGB1-dependent route that glycyrrhizin blocked, damping caspase-11-dependent immune responses and organ injury [52].

### 4.2. HMGB1 in Organs Affected by Immune-Related Adverse Events

Serum HMGB1 predicted checkpoint inhibitor pneumonitis at two university hospitals [53], and a prospective multicentre study then evaluated it for risk assessment of severe pneumonitis [54]. A murine heart model combining radiotherapy with programmed cell death protein 1 (PD-1) blockade sustained more myocardial injury than radiotherapy alone, with rises in myocardial HMGB1, IL-1β and IL-18, increased fibrosis and upregulation of the HMGB1–caspase-1–gasdermin D (GSDMD) pyroptosis axis [55]. The liver has the weakest direct support: HMGB1 contributes to acute allograft rejection by promoting dendritic cell maturation and T-cell proliferation [56]—the same dendritic cell–T-cell axis that checkpoint blockade unleashes, but a transplant model rather than checkpoint inhibitor hepatotoxicity. Colitis and autoimmune thyroiditis rest on preclinical work [57,58].

Glycyrrhizic acid class compounds themselves protect organs through this axis, though mostly outside oncology: glycyrrhizin inhibited HMGB1-mediated ferroptosis in renal tubular epithelial cells in contrast-induced acute kidney injury [59], streptozotocin-diabetic rats given it showed less kidney injury [60], HMGB1 also mediates podocyte injury in a nicotine-exposed podocyte model [61], and in a cardiac-arrest model glycyrrhizin-mediated HMGB1 inhibition blunted rises in HMGB1, IL-6 and IL-10 and was associated with reduced cerebral T-cell infiltration and better neurological outcome [62].

Taken together, these findings support the proposition that HMGB1 participates in the sterile inflammation underlying irAEs, but the strength of that evidence differs by organ: clinical and prospective for pneumonitis, direct but preclinical and single-model for the heart, and indirect for the liver, gut and thyroid, where the models reproduce the organ disease rather than checkpoint inhibitor toxicity. The liver is the hard case: it carries the strongest clinical data on this class, yet its HMGB1 evidence comes from models other than checkpoint inhibitor hepatotoxicity.

### 4.3. HMGB1 in the Tumor Microenvironment

Whether extracellular HMGB1 is immunostimulatory or immunosuppressive is not fixed: three mutually exclusive redox forms have different functions—reduced HMGB1 is chemotactic, disulfide HMGB1 is pro-inflammatory and drives cytokine release through Toll-like receptor 4 (TLR4), and HMGB1 oxidized at Cys-106 has lost its immunostimulatory activity [47,48]—and which form predominates depends on the compartment and on the oxidative environment. In the dendritic-cell system of Hubert et al. [63], terminally oxidized HMGB1 additionally signaled tolerance through RAGE; whether it holds in other cell types or settings is not resolved by the studies identified here.

Within the tumor microenvironment, the balance of evidence is toward immunosuppression. In sepsis, signaling through Toll-like receptor 2 (TLR2) raises programmed death-ligand 1 (PD-L1) on neutrophils and leads to T-cell apoptosis [64]—shown in that setting rather than in a tumor; tumor models instead show expansion of myeloid-derived suppressor cells (MDSCs) and immune escape [65], and in melanoma it is TLR4 signaling that drives myeloid-derived suppressor cell (MDSC)-mediated immunosuppression [66]. In murine breast-tumor models, Hubert et al. linked extracellular HMGB1 signaling to an immunosuppressive microenvironment: a substantial fraction of tumor-interstitial HMGB1 was oxidized, and oxidized HMGB1 acted as a tolerogenic signal through RAGE in that system [63]. Their checkpoint-combination experiments used a RAGE antagonist peptide and ethyl pyruvate rather than a compound of this class, and the antitumour gains were model-dependent, with the anti-PD-1 effect in the 4T1 model not reaching statistical significance. This part of the tumor-compartment hypothesis is supported by how the oxidized form behaved in that dendritic-cell system; the other tumor studies reviewed here do not uniformly identify the relevant redox form. In non-small cell lung cancer, pretreatment tumor HMGB1 mRNA was higher in patients without clinical benefit from checkpoint blockade, while plasma HMGB1 protein did not distinguish patients with and without benefit [67]: the clinical association concerns tissue transcript, not the circulating protein, and neither measurement identifies the extracellular redox-active pool or shows a therapeutic effect of HMGB1 blockade. Inhibiting this axis may therefore be desirable for antitumour immunity and protective against immune-mediated tissue injury at once—but only if the blockade reaches the form that matters in each compartment.

HMGB1 also supports the antitumour response, and the tumor-compartment argument has to accommodate that. In murine tumor models, HMGB1–TLR4 signaling on dendritic cells was required for the antigen handling that produced effective antitumour immune responses after chemotherapy or radiotherapy [21], and the chemotactic and pro-inflammatory activities of HMGB1 are carried by mutually exclusive redox forms rather than by a graded amount of one molecule [20]. Recruiting antigen-presenting cells and sustaining their antigen-presenting function are separate steps, so sparing one HMGB1 function would not preserve every beneficial effect, and the fully reduced, chemotactic form participates in sterile inflammation as well. Any proposed benefit therefore depends on the compartment, the molecular form, the treatment schedule and the tumor model, and it requires simultaneous assessment of organ injury and tumor control.

### 4.4. Why the Two Directions Are Compatible

This convergence yields the central hypothesis of this review. If HMGB1 acts as a sterile-inflammation damage-associated molecular pattern in the organs that sustain irAEs, and as an immunosuppressive mediator within the tumor microenvironment, then a blockade reaching the relevant form in each compartment might attenuate irAE severity without necessarily costing the antitumour response. That is a conditional prediction rather than a demonstrated reconciliation: the compartment split alone does not deliver it, and the same protein also supports antitumour immune priming in treatment-specific settings (Section 4.3). Compartment, redox form, the inside/outside distinction and the timing of release are four separate axes on which the net effect may depend; redox-form selectivity would be one way for the framework to work, but the evidence reviewed here does not establish it as a necessary condition, and whether any member of this class has such selectivity remains to be tested.

### 4.5. The Supporting Experiment and Its Limits

In tumor-bearing mice receiving radiotherapy together with PD-1 blockade, a neutralizing HMGB1 antibody reduced histological and biochemical myocardial injury—interstitial collagen volume fraction fell from 4.16% to 1.94% (*p* < 0.0001), with lower myocardial HMGB1, IL-1β and IL-18 and lower creatine kinase (CK) and its MB isoform (CK-MB) (*p* < 0.05, values not reported)—and down-regulated the HMGB1–caspase-1–GSDMD pyroptosis pathway, while ejection fraction and fractional shortening did not improve [55]. In the same mice, day-21 tumor volume and weight did not differ significantly between the antibody-treated and the combined-treatment-only arms (*p* > 0.05, n = 5 per group), and peripheral blood lymphocyte distribution was similar (n = 3); the study reports no equivalence margin and no power analysis, so this is a null result rather than a demonstration that antitumour efficacy was preserved.

The support is therefore narrow and singular: one organ, one model, one agent—and an agent that is not a member of this class. Four further limits bear on how much it can carry. The antibody was given once, 20 µg intraperitoneally on day 15, two days after the last radiotherapy and PD-1 dose: a delayed-intervention design, which the study did not compare against early or concurrent blockade. There was no isotype control and no antibody-alone arm, which limits attribution of the cardiac effect to HMGB1 neutralization and prevents an interaction analysis between antibody treatment and the radiotherapy-plus-PD-1 schedule. The two-group comparison can estimate the incremental tumor-control effect of adding the antibody, but its precision is limited by the small sample and the short follow-up. The tumor comparisons rest on five mice per group and the peripheral-blood and pathway data on three, and body weights were recorded but not reported, leaving no systemic-toxicity readout. The agent was a neutralizing antibody, so the findings do not directly establish efficacy for MgIG or glycyrrhizin, and the organ was the heart, injured by radiotherapy, rather than the liver. Whether an HMGB1-binding small molecule at a well-tolerated dose reproduces the antibody’s effect in a liver-directed model is untested but answerable, and Section 8 sets out the predictions that would falsify the framework.

## 5. Antitumour Immunity and the Glycyrrhizic Acid Class

The glycyrrhizic acid class acts on antitumour immunity indirectly: it reduces the cell populations that suppress antitumour responses and can induce immunogenic cell death. Constituents of the same plant that are not members of this class are considered separately in Section 5.2. Table S2 tabulates compound-specific preclinical studies with their models, routes of administration and the stated isomer identity of each source; it is a working table rather than an exhaustive census, and the sources it omits are cited in the text.

### 5.1. Triterpenoid Constituents Remodel the Immunosuppressive Microenvironment

The clearest demonstration is in melanoma, where glycyrrhizic acid restricted tumor progression, correlating with a shift from Th2 to Th1 cytokines and with attenuation of regulatory T cells and myeloid-derived suppressor cells [68]. A GA-modified prodrug increased CD4^+^ and CD8^+^ T cells at the tumor site and reduced regulatory T-cell and Th2 subsets [69].

Immunogenic cell death is a second proposed route: GA triggered endoplasmic reticulum stress–pyroptosis crosstalk in hepatocellular carcinoma cells, eliciting immunogenic cell death in a tumor type whose immunosuppressive microenvironment obstructs immunotherapy [70]. This is the tension developed in Section 6 in a different form, because immunogenic cell death releases HMGB1 as one of its markers [70,71], yet sustained extracellular HMGB1 can be immunosuppressive: the chemotactic and pro-inflammatory activities belong to mutually exclusive redox forms, the chemotactic one also contributes to sterile inflammation, and in the dendritic-cell system studied by Hubert et al. oxidized HMGB1 had RAGE-dependent tolerogenic activity, whereas the other tumor studies reviewed here do not uniformly identify the relevant redox form (Section 4.3) [20,47,48,63]. Whether the net effect of a member of this class is helpful or harmful may therefore depend on the balance between these forms, on when the compound is present relative to HMGB1 release, on how much reaches each tissue, and on its other targets; whether any member has a useful preference among the forms is untested, and such a preference would be a route to optimization rather than a condition the class must satisfy.

### 5.2. Adjacent Constituents of Glycyrrhiza: Evidence That Does Not Belong to This Class

Several constituents of the same plant are routinely discussed alongside this class but are different chemical entities: chalcones (licochalcone A, isoliquiritigenin), a flavanone glycoside (liquiritin), isoflavonoids (glabridin, licoricidin), polysaccharides, extracts and multi-herb formulas. They are collected for completeness and are not used to support any conclusion about this class; several of them have been reviewed elsewhere [10,11].

Direct modulation of checkpoint molecules—ligand binding or expression—is reported for these adjacent constituents rather than for the triterpenoids, which instead show changes in checkpoint phenotype in immune cells as a secondary consequence of microenvironment remodeling (Section 4.3). In colon cancer cells licochalcone A acted on PD-L1 through nuclear factor κB (NF-κB) and Ras/Raf/MEK [72], and in lung cancer cells it lowered PD-L1 after induction by interferon-γ [73]. Isoliquiritigenin reduced PD-L1 and tumor progression in melanoma by inhibiting STAT3 phosphorylation [74], enhanced host antitumour immunity in breast tumors [75], and reduced gastric cancer stemness by downregulating GRP78 [76]; *G. uralensis* phenolics inhibited PD-1/PD-L1 only weakly (30–65% at 100 µM) [77]; liquiritin reduced breast-cancer neoangiogenesis through tumor-associated macrophages [78]; licoricidin released HMGB1 and ATP from cervical cancer cells [71]; *Glycyrrhiza polysaccharides* increased IFN-γ-producing intestinal γδT cells through the microbiota and mucosal TLR/NF-κB signaling [79] and serve as vaccine adjuvants [80,81]; and a glabridin–gold(I) complex suppressed tumor-cell PD-L1 in liver cancer models [82].

Evidence at the level of extracts and formulas is weaker again, because the active constituent cannot be identified at all. In non-small cell lung cancer a licorice extract slowed growth, lowering the CDK4–cyclin D1 complex and bringing in more CD8^+^ T cells [83], and the licorice-containing formula Gancao Ganjiang decoction limited bleomycin-induced pulmonary fibrosis by way of a PD-1/TGF-β1/IL-17A pathway [84].

One finding in this group cuts the other way. In experimental autoimmune encephalomyelitis, licochalcone A suppressed hydrogen peroxide, nitric oxide, IFN-γ, TNF-α and IL-17 production from splenocytes, and three of those cytokines from peritoneal cells [85]—the opposite direction to its antitumour effects. It concerns a chalcone rather than a member of this class, but shows that chemically distinct constituents of one plant can move immunity in opposite directions.

### 5.3. The Clinical Gap in This Stream

The evidence for the class itself is preclinical. Within the limits of our search (Section 2.3), no human study isolates the effect of a glycyrrhizic acid class compound on PD-1 or PD-L1 expression, because the one clinical comparison that measured these outcomes gave MgIG to both arms [43]; direct checkpoint modulation in the licorice literature belongs to the constituents of Section 5.2. The one clinical study that measured peripheral blood PD-1 and PD-L1 in a relevant population compared MgIG alone with MgIG plus tripterygium glycosides in autoimmune hepatitis, so the comparison estimates the effect of adding tripterygium glycosides to MgIG; it cannot isolate the effect of MgIG itself [43]; it is informative about the clinical use of this class in immune-mediated liver disease (Section 7.3) but cannot support checkpoint modulation by the class itself. Whether triterpenoid immunostimulation adds to, or merely overlaps with, the effects of checkpoint blockade is untested.

A further complication is that the sign of any immune effect depends on the compartment: in oncology, downregulation of PD-L1 in tumor cells is expected to be favorable, whereas the same molecular event in an inflammatory compartment cannot be read the same way, because PD-1/PD-L1 signaling restrains T-cell effector function and thereby limits tissue damage. Lowering checkpoint expression may thus be desirable in one compartment and harmful in another—a tension revisited in Section 9.

## 6. Glycyrrhizic Acid Class Compounds as Delivery Platforms

A second, largely separate literature has explored this class as an adjunct to—or vehicle for—cancer immunotherapy. Its defining feature is that the glycyrrhizic acid moiety is rarely a passive excipient: it contributes self-assembly, hepatic targeting reported for glycyrrhetinic acid derivatives, and in several designs an immunological function of its own.

### 6.1. Platform Designs That Exploit an Intrinsic Immune Function

The design most directly relevant to this review’s argument combines ferroptosis induction with HMGB1 blockade. Because ferroptotic tumor cell death recruits myeloid-derived suppressor cells through HMGB1 secretion, offsetting the immunogenicity of the death itself, a nanoplatform was built to deliver glycyrrhizic acid alongside a glutathione-depleting, iron-generating core, aiming to elicit systemic antitumour immunity while preventing the MDSC response [86]. It is the closest published instantiation of the framework’s tumor-compartment prediction using a member of this class.

Other platforms use glycyrrhizic acid as a functional scaffold: a nanotheranostic system suppressing heat-shock protein 90 (HSP90)-mediated thermoresistance in hepatocellular carcinoma [87], an alginate nanogel co-delivering glycyrrhizin with doxorubicin [88], and GA nanoparticles combined with ferrotherapy [89]. Further designs are cataloged elsewhere [90].

### 6.2. Combination Designs

Several studies pair a member of this class with a second immunologically active element: a metformin–GA hydrogel producing synergistic immunotherapy through spatiotemporal remodeling of the microenvironment [91], glycyrrhizic acid-loaded hybrid liposomes designed to target both inflammatory cascades and PD-1/PD-L1, though in a model of neuroinflammation rather than cancer [92], and GA with doxorubicin in a redox-sensitive nanosystem [93]; related nano-herb strategies have been developed for multiple tumor immune activation [94].

### 6.3. Limits of the Platform Designs

These studies are better characterized as engineering than as pharmacology: the glycyrrhizic acid moiety frequently serves simultaneously as carrier, targeting ligand and active agent, and its contribution is rarely dissected from the payload by appropriate controls. They establish the feasibility of combining this class with immunotherapy but do not show that the class contributes antitumour immunity in patients: no agent from this stream has been taken into an ICI trial identified here, and the designs tested alongside checkpoint blockade report antitumour rather than irAE endpoints [89].

A further caution follows from Section 5.1. Triggers of immunogenic cell death depend on HMGB1 release to recruit antigen-presenting cells, so a delivery system that induces immunogenic cell death while blocking HMGB1 cannot solve the problem by confinement to the extracellular compartment: the recruiting signal is itself extracellular, and it is the reduced form. Redox-form selectivity—sparing reduced HMGB1 while blocking the disulfide form—or timing, releasing HMGB1 first and blocking afterward, could separate the two roles; neither has been shown to account for the protection observed with a member of this class, and neither would preserve every beneficial HMGB1 function (Section 4.3). The ferroptosis-plus-HMGB1-blockade platform is the best-controlled case in this stream, in which the increment attributable to the compound is measured rather than inferred [86]; its controls, endpoints, what it does not establish and its single-model limit are set out in Section S4, and no claim of prolonged survival is taken from it.

## 7. Protection Against Immune-Mediated Organ Injury

### 7.1. Clinical Evidence

The most direct evidence concerns the liver. A multicentre retrospective case–control study with propensity-score matching screened 359,460 hospitalized cases across five tertiary hospitals, identified 318 confirmed or highly likely cases of ChILI by expert review, and matched 229 (114 receiving MgIG plus steroid, 115 steroid alone) [1]. The predominant causative agents were camrelizumab, sintilimab and tislelizumab; the median MgIG dose was 200 mg/day for a median of 8 days.

Table S1 sets out the Zhi 2025 cohort row by row, each row stating its own time point; not all are day-10 measurements. The last two rows of Table 1 are context rather than oncology evidence: the 200 versus 100 mg/day comparison was made in chronic liver disease, and the autoimmune-hepatitis trial isolates the added immunosuppressant rather than the class compound.

Among patients with high-grade injury (CTCAE grade 3–4; 93 versus 89), an average daily methylprednisolone-equivalent dose of ≥1.5 mg/kg over the recorded steroid course (cumulative dose ÷ steroid days ÷ body weight) was less frequent in the combination group (1 of 93, 1.1%, versus 8 of 89, 9.0%; *p* = 0.017) [1]. Whole-cohort counts did not reach significance (2 of 114 versus 9 of 115; *p* = 0.059), so this is a subgroup finding, and a lower average daily dose category is not a lower cumulative dose or a lower systemic exposure (Section 9.3). Because early steroid exposure for irAEs has itself been associated with a smaller survival benefit [2], requiring less steroid could bear on antitumour efficacy, although the Zhi 2025 cohort did not report a survival endpoint (Section 10). Total length of stay was also shorter, 15 versus 17 days (*p* = 0.020).

The prevention data come from a single arm: sixty-seven patients with advanced cancer beginning ICI-based regimens received prophylactic MgIG (200 mg intravenously, ≥3 consecutive days within ±2 days of each cycle, across a planned 4–6 cycles). All 67 received an ICI-containing combination regimen (50 with chemotherapy, 3 with a targeted agent and 14 with both), and 27 (40.3%) also received glucocorticoid premedication, mainly dexamethasone. Liver injury was defined as any of ALT, AST, alkaline phosphatase (ALP) or total bilirubin above the upper limit of normal in patients without hepatocellular carcinoma or liver metastases, and by higher thresholds (ALT or AST > 3×, ALP > 2.5×, bilirubin > 1.5×) in patients with either, graded by CTCAE version 5.0 before each cycle. Six of the 67 developed liver injury, an incidence of 9.0% (95% CI 3.4–18.5%); in the per-protocol subset of 53 patients who completed 4–6 cycles it was 3.8% (2/53, 95% CI 0.5–13.0%), against a failure threshold reported as prespecified (upper confidence limit above 20%). That subset excluded some patients who had already developed liver injury—14 patients stopped after 1–3 cycles for reasons that included progression, loss to follow-up and early liver injury—so the lower per-protocol incidence does not provide independent support; injuries were predominantly grade I–II, no grade IV events occurred, and no serious adverse event was attributed to MgIG [5]. Reported incidences in Chinese patients receiving combination ICI regimens range from 11.2% to 36.5% [5], so the observed 9.0% falls below that range. Those historical rates were obtained under different regimens and definitions; they do not provide a valid concurrent comparison for estimating prophylactic efficacy.

Baseline total bilirubin was lower in the six patients who went on to develop liver injury (6.8 [4.6–10.3] versus 11.2 [8.2–13.3] µmol/L, *p* < 0.05), and the authors propose that low baseline bilirubin may mark insufficient antioxidant reserve [5]. With six events and no adjustment for multiple testing, this is an exploratory observation rather than a validated predictive marker, and it does not displace studies identifying sex, underlying liver disease and concomitant medication as the principal risk factors.

Three further studies were done in chemotherapy-treated populations. MAGIC-301 was a prospective, open-label, randomized multicentre trial at 28 centers in China (2013–2015): 1200 patients receiving chemotherapy of defined hepatotoxic risk were randomized 2:1 to MgIG 200 mg/day from the day before chemotherapy for at least 5 days or to chemotherapy alone, graded by Common Terminology Criteria for Adverse Events version 4.0. Among the 1146 in the full analysis set, liver injury fell from 62.63% to 52.81% (reported *p* = 0.0004; the recomputation from the published counts is in Section S3); the overall grade distribution shifted in favor of MgIG (Wilcoxon *p* = 0.0008; graded counts 765 versus 380), while the grade ≥2 rate in those counts was 10.07% (77 of 765) versus 12.37% (47 of 380), a descriptive proportion that is not the endpoint the reported *p* value describes; ALT and AST abnormalities at the day 13–15 visit were 8.63% and 10.72% versus 16.58% and 20.05% (both *p* < 0.0001); and rescue hepatoprotective treatment was required in 9.2% of controls versus 1.3% (*p* < 0.0001) (Table 1) [95]. A second randomized trial in 216 treatment-naive patients with gastrointestinal malignancies receiving FOLFOX or XELOX allocated them to prophylactic MgIG (n = 114) or glutathione (n = 102) and reported abnormal liver biochemistry in 12 versus 28 courses (*p* < 0.01), with no grade II or higher hepatotoxicity in either arm [96]; that comparison is against another hepatoprotectant, its allocation concealment and blinding are not described, and it is a chemotherapy rather than an ICI population, which this review does not appraise systematically.

The third study was an uncontrolled before-after observation: 1511 of 1567 patients with hematologic malignancies received prophylactic MgIG, and liver biochemical indices were not increased after chemotherapy relative to baseline [97]. Without a control group it cannot separate prophylaxis from the natural history of chemotherapy-associated liver injury, so the observation is compatible with no treatment effect.

The MAGIC-301 endpoint is a liver biochemical abnormality within that trial’s defined visit window in a chemotherapy-treated population, not an independently adjudicated immune-mediated hepatitis, and its grade ≥2 rate is reported above as a descriptive proportion. None of the three chemotherapy studies addresses checkpoint inhibitor-associated injury, and none measured antitumour efficacy; the two ChILI datasets are confined to the liver, so protection of other irAE-affected organs remains untested clinically. One conference abstract on MgIG in liver injury from novel antitumour drugs is cited by a recent review [4] but is not tabulated here.

### 7.2. Evidence in T-Cell-Mediated Liver Injury

Behind the clinical findings lies a substantial preclinical literature, most of it in models whose effector mechanism is T-cell-mediated—the mechanism of checkpoint inhibitor hepatotoxicity.

The preclinical liver-injury studies are detailed in Section S4 and Table S2: MgIG in ConA models [18,19,44,46], other glycyrrhizic acid class compounds in ConA models [98,99,100], and an abstract-level MgIG study using a D-galactosamine/Freund’s complete adjuvant model [16]. A separate study examined glycyrrhizin against renal inflammation in mice with ConA hepatitis [101]. The clearest English-language statement of the product’s configuration comes from the MgIG study of Yang et al. [44] (Section 3.3).

Two studies tie the class to the HMGB1 arm directly: in an α-naphthyl isothiocyanate model, GA ameliorated cholestatic liver injury by inhibiting acetylated HMGB1/TLR2-mediated crosstalk between hepatocytes and macrophages [102], placing an HMGB1-dependent mechanism inside the phenotype FXR/PXR signaling would be expected to address; and glycyrrhizin lessened hepatic ischemia-reperfusion injury through HMGB1-dependent, GSDMD-mediated pyroptosis of Kupffer cells [103], the axis up-regulated in the cardiac injury model of radiotherapy plus PD-1 blockade (Section 4.5). A third hepatic mechanism runs through PXR: 18β-GA prevented D-galactosamine/lipopolysaccharide-induced liver injury by inhibiting autophagic degradation through that receptor [104].

The class has also been tested outside the organ that was injured: in a mouse ConA hepatitis model, glycyrrhizin attenuated renal inflammation through the IL-25/M2 axis [101]—the closest published precedent for the cross-organ proposition of Section 4, though a single non-oncological study (Section S4).

How far the model can be pushed is a separate question. The ConA model is a legitimate but imperfect bridge to ChILI: it was originally described as a T-cell-dependent injury requiring both T lymphocytes and macrophages [105], and its biochemistry and histology resemble human viral and autoimmune liver disease more closely than carbon tetrachloride or D-galactosamine models do. ConA, however, activates T cells directly as a lectin mitogen, whereas checkpoint inhibitor hepatitis arises when checkpoint blockade releases pre-existing autoreactive T cells from inhibition, so transfer to the clinic cannot be assumed.

### 7.3. Other Organs, and an Attribution Problem

Attribution is a problem even inside the liver: in autoimmune hepatitis, adding tripterygium glycosides to MgIG improved liver biochemistry and reduced peripheral blood PD-1 and PD-L1 expression together with serum IFN-γ [43], but the comparator was MgIG alone, so the checkpoint and cytokine changes cannot be attributed to the class agent (Section 5.3).

Outside the liver the evidence is thinner: glycyrrhizic acid attenuated injury in streptozotocin-induced diabetic rats [60] and HMGB1 was shown to mediate podocyte injury [61], and a licorice-containing formula reduced acute lung injury through the PD-1/IL-17A pathway [106], though that intervention is a multi-herb formula rather than a defined compound.

The preclinical literature thus supports organ protection in more than one tissue, but the studies differ in compound, model, species and publication language, and the two most directly relevant clinical datasets both concern the liver. The isomer is unstated in most of these reports—the empirical basis of the recommendation in Section 11.5—so when this literature is cited as support for the mechanism proposed in Section 4, it is not always possible to say which agent was tested.

## 8. Testing the HMGB1 Hypothesis Against the Assembled Evidence

Section 4 proposed that HMGB1 blockade explains both the organ-protective and putative immunoenhancing effects of this class. 

**Figure 1:**
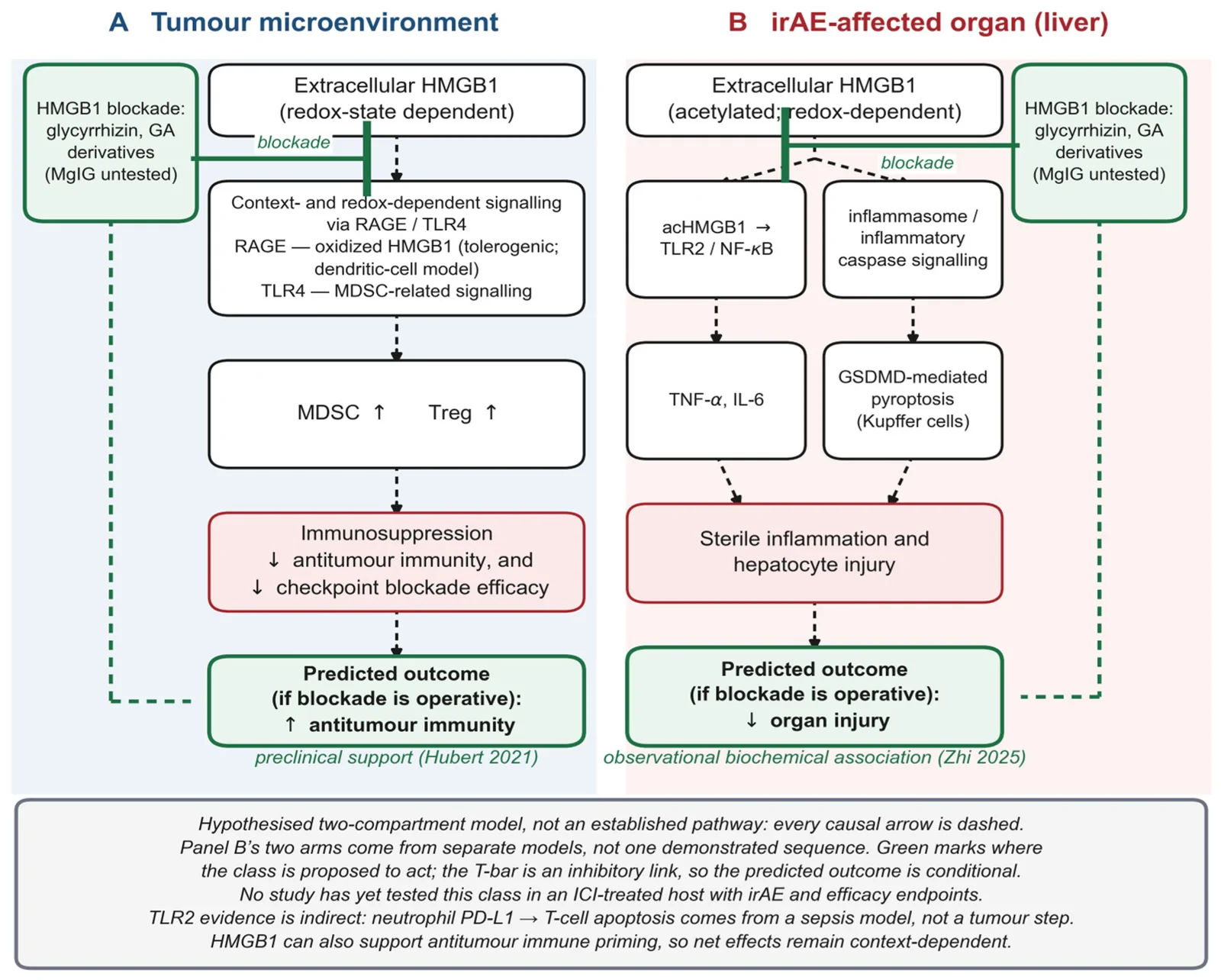
Compartment-specific HMGB1 blockade as a proposed compartment-dependent mechanism. In the tumor microenvironment (**A**), extracellular HMGB1 promotes myeloid-derived suppressor cell and regulatory T-cell populations, producing immunosuppression that limits checkpoint blockade efficacy; neutrophil PD-L1 expression with T-cell apoptosis is summarized in the figure note as an indirect clue from a non-tumor (sepsis) model [63], not as a step verified in a tumor. In an irAE-affected organ such as the liver (**B**), two parallel proposals are shown rather than one demonstrated sequence: acetylated HMGB1 signaling through TLR2/NF-κB, and HMGB1-dependent inflammasome and caspase signaling leading to GSDMD-mediated pyroptosis in Kupffer cells; the figure draws no conclusion about RAGE or TLR4 in that compartment. The two compartments are proposed to pull in opposite directions, so a single pharmacological action—blockade by glycyrrhizic acid class compounds—is predicted to help in both, and only to the extent that it reaches the relevant form in each. Panel A rests on tumor models that tested several HMGB1-directed agents—glycyrrhizin, the RAGE antagonist peptide, ethyl pyruvate and the HMGB1 A box protein (the N-terminal DNA-binding domain)—and the experiments combined with checkpoint blockade used the peptide and ethyl pyruvate rather than a class compound, so that evidence cannot be read as evidence about MgIG. Panel B combines mechanistic observations from non-ICI liver-injury models with an observational association between MgIG and biochemical recovery in ChILI [1]; the clinical study did not establish HMGB1 involvement. No study identified here has tested the convergent prediction for any member of this class in an ICI-treated tumor-bearing host. Dashed arrows denote links that are proposed rather than observed, green marks where the class is proposed to act, and the T-bar is an inhibitory link. Abbreviations: HMGB1, high-mobility group box 1; RAGE, receptor for advanced glycation end-products; TLR2/TLR4, Toll-like receptors 2 and 4; MDSC, myeloid-derived suppressor cell; Treg, regulatory T-cell; PD-L1, programmed death-ligand 1; irAE, immune-related adverse event; MgIG, magnesium isoglycyrrhizinate; GA, glycyrrhetinic acid; ICI, immune checkpoint inhibitor.

Figure 1 sets out the framework as a two-compartment model—extracellular HMGB1 sustains immunosuppression within the tumor microenvironment (panel A) while driving sterile inflammation in irAE-affected organs (panel B)—in which a single pharmacological action is proposed to be beneficial in both, conditionally on the form of HMGB1 it reaches and on the schedule that delivers it. Three qualifications apply. It does not distinguish the HMGB1 redox forms, which have different functions (Section 4.3 and Section 5.1). Its receptors rest on evidence of different kinds: in the tumor compartment RAGE carries the tolerogenic signal of oxidized HMGB1 [63], TLR4 the myeloid-suppressor evidence [66], and TLR2 the neutrophil PD-L1 evidence, which comes from sepsis rather than a tumor [64]; in the liver, two parallel proposals from two different models—acHMGB1/TLR2/NF-κB [102] and HMGB1-dependent GSDMD-mediated pyroptosis [103]—are shown side by side rather than as a demonstrated sequence. And HMGB1 also supports antitumour immune priming in treatment-specific settings (Section 4.3), so a net benefit is a prediction of the model rather than a consequence of it. No experiment has yet combined a member of this class, an ICI-treated tumor-bearing host and both irAE and efficacy endpoints; the closest published result tests an antibody rather than this class (Section 4.5).

Prediction 1—the hepatoprotective benefit should be larger where circulating HMGB1 is higher, and the test is an interaction rather than a correlation. The marker is measurable and prognostically informative: serum HMGB1 has been proposed as a marker whose fall reflects the anti-inflammatory effect of glycyrrhizin in drug-induced liver injury [4], and circulating HMGB1 predicts checkpoint inhibitor pneumonitis [53,54]. Prognostic is not predictive: a marker of injury severity will correlate with recovery whether or not the treatment works, and separating the two requires a comparator arm, pre-treatment sampling and a formal interaction test. Only the matched cohort has a comparator [1], pre-treatment serum is not documented in either publication, and the single-arm prevention study [5] cannot show a differential effect.

Prediction 2—if the liver limb is HMGB1-dependent, protection should track HMGB1 binding at matched isomer concentrations, and should be lost when the HMGB1 pathway is removed. The evidence is compatible but not decisive, and the obvious test does not discriminate, because comparing an 18α with an 18β preparation confounds stereochemistry with absorption, tissue exposure and other targets. Glycyrrhizin, whose aglycone is 18β, is the canonical small-molecule HMGB1 inhibitor [50] and protects organs in non-oncological models [60]; MgIG, the 18α preparation used clinically, is reported to protect the liver in the two checkpoint-inhibitor datasets [1,5] and reduced chemotherapy-associated liver injury in a randomized trial [95]; no study identified here compares their binding affinities at matched concentrations. The matched-concentration comparison and the loss-of-function test specified in Section 12 would settle it; a protection that substantially survives would identify an HMGB1-independent component whose identity could not be assigned to 11β-HSD1 [23,32], to the FXR/PXR–bile-acid arm [14] or to any other target without separate experiments.

Prediction 3—protection should track HMGB1 dependence across irAE types. The organ-level evidence lies outside immune-related toxicity. In the kidney, glycyrrhizic acid protected diabetic rats explicitly as an HMGB1 inhibitor [60], which shows only that HMGB1-directed protection is attainable in a second organ, since diabetic nephropathy is not an irAE. The lung does not test it cleanly: the only lung-injury study identified here uses a multi-herb formula and reports no HMGB1 measurement [106]. In the liver, where the class’s clinical evidence is strongest, the prediction cannot be tested with the data assembled here, because HMGB1 involvement in ChILI is uncharacterized.

**Figure 2:**
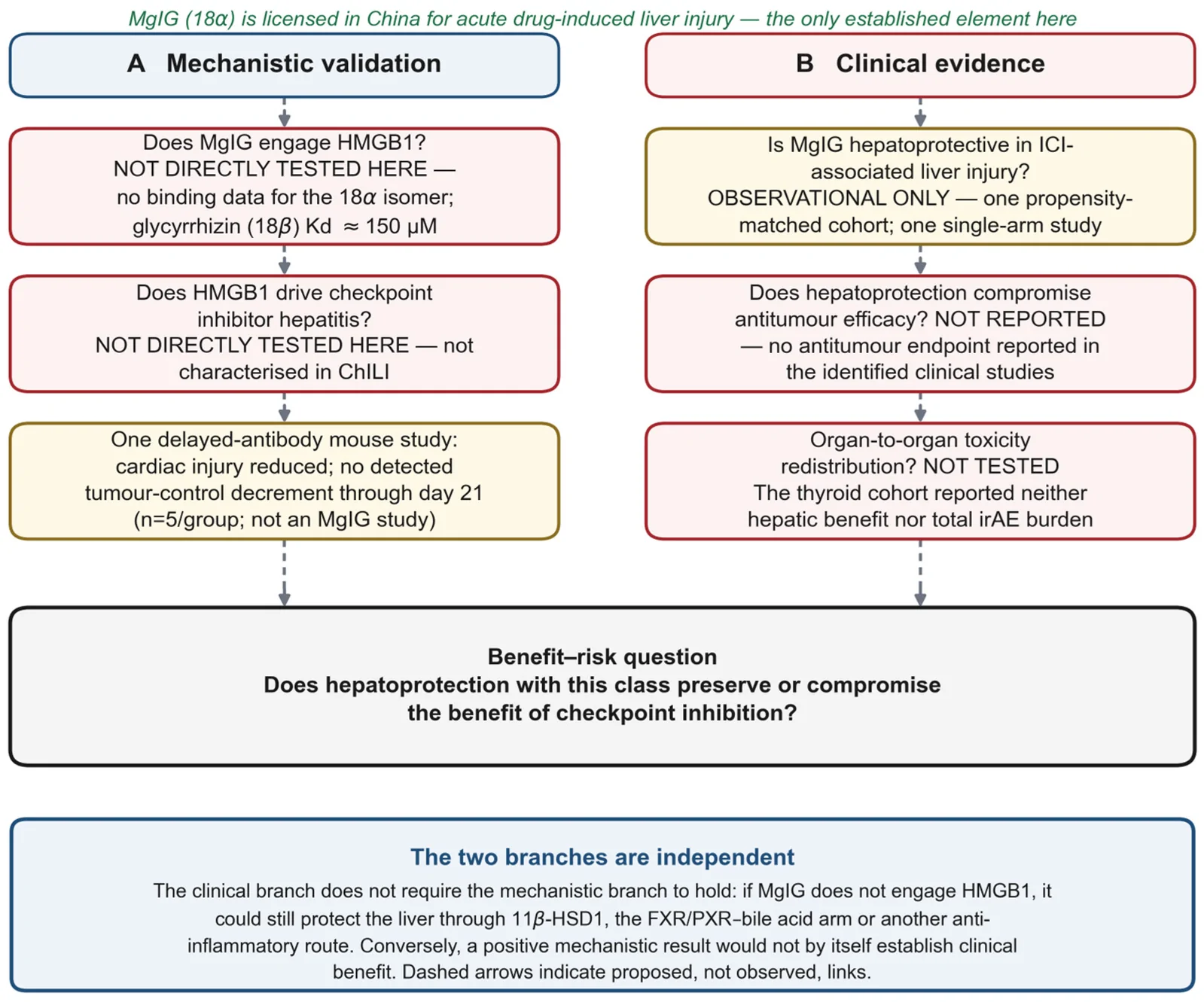
Evidence status of the proposed chain from magnesium isoglycyrrhizinate to clinical benefit. Each step is annotated with the strongest evidence identified for it: licensed use (MgIG’s approval for acute drug-induced liver injury in China, a product fact); not directly tested in the identified literature (binding of HMGB1 by the 18α isomer; a causal role for HMGB1 in checkpoint inhibitor hepatitis); one delayed-antibody mouse study (cardiac injury reduced; no detected tumor-control decrement through day 21; five mice per group; an antibody outside this class, not a test of MgIG); observational only (hepatoprotection in checkpoint inhibitor hepatitis, from one propensity-matched cohort and one single-arm prevention study); and not reported (progression-free and overall survival were not among the reported outcomes of the clinical studies identified here). The figure summarizes evidence status and is not a treatment algorithm; prophylactic use, unstandardized licorice products and use without potassium monitoring are not supported by current evidence. Dashed arrows denote links that are proposed rather than observed. Abbreviations: MgIG, magnesium isoglycyrrhizinate; ChILI, checkpoint inhibitor-induced liver injury; ICI, immune checkpoint inhibitor; irAE, immune-related adverse event; ALT, alanine aminotransferase; HMGB1, high-mobility group box 1; PD-1/PD-L1, programmed cell death protein 1 and its ligand.

Assessment. The hypothesis is coherent, but its two mechanistic premises are unverified—that MgIG engages HMGB1, and that HMGB1 contributes materially to ChILI—and no study identified here has tested either. Whether protecting the organ comes at the cost of antitumour immunity is not a third premise but a therapeutic outcome that no study identified here has measured, and only a design carrying both endpoint families can answer it, which is why Figure 2 separates that benefit–risk question from the mechanistic ones.

Do the two mechanistic arms interact? The HMGB1 axis of Section 4 and the FXR/PXR arm of Section 3.2 were developed in parallel, but one study places an HMGB1-dependent mechanism inside the cholestatic phenotype that FXR/PXR signaling governs: in α-naphthyl isothiocyanate injury, GA inhibited acetylated HMGB1/TLR2 crosstalk between hepatocytes and macrophages [102]. The two could run in either direction, or converge on a shared terminal event—Kupffer cell pyroptosis, for which HMGB1-dependent, GSDMD-mediated signaling has been shown with glycyrrhizin [103]. If the nuclear receptors act upstream, the class could protect the liver without engaging HMGB1 at all.

## 9. The Immune-Safety Question

### 9.1. The Tension

Section 5, Section 6, Section 7 and Section 8 describe a class that appears to protect the liver in ICI-treated patients, remodels immunosuppressive cell populations, and has been engineered into platforms intended to amplify antitumour immunity; separately, adjacent *Glycyrrhiza* constituents—licochalcone A and isoliquiritigenin among them—have been reported to modulate checkpoint molecules (Section 5.2). A compartment-dependent effect is one possible explanation for these observations, although distinct targets or mechanisms could also contribute. These observations would point in the same direction if the underlying mechanism were compartment-specific; if the anti-inflammatory action that protects the liver also suppresses the antitumour response, hepatoprotection with this class could compromise the very therapy it is meant to support.

This is not a hypothetical worry. Corticosteroids, the standard of care for irAEs, illustrate the difficulty: in the same 20,163-patient cohort described in Section 1, what tracked with poorer outcome was timing rather than steroid use itself [2]. A hepatoprotective agent whose anti-inflammatory action suppressed the antitumour response would raise the same problem.

### 9.2. Evidence Pointing to Harm: A Potential Thyroid Safety Signal

The most direct safety signal concerns neither antitumour efficacy nor the liver, but the thyroid. A single-center observational (real-world) cohort followed 175 patients with normal thyroid function starting PD-1 inhibitor therapy between January 2021 and July 2023; 62 received prophylactic glycyrrhizic acid class hepatoprotectants and 113 did not, with emergence of thyroid dysfunction as the outcome [107].

The difference in timing was substantial. In the unweighted Kaplan–Meier analysis, the time to 50% cumulative incidence of thyroid dysfunction was 2.5 months (95% CI 1.47–3.53) in the treated group versus 8 months (95% CI 5.96–10.04) in controls (*p* < 0.001)—a time to a cumulative-incidence threshold, not the observed median time to onset of thyroid dysfunction in either group. In multivariable Cox regression with variables selected by Lasso, the use of a glycyrrhizic acid class hepatoprotectant was associated with thyroid dysfunction in the adjusted model (hazard ratio (HR) 1.774, 95% CI 1.032–3.051, *p* = 0.038), with no formulation-specific effect estimates reported, alongside gastrointestinal tumors (HR 2.223, 95% CI 1.096–4.509), other tumor types (HR 2.320, 95% CI 1.093–4.920) and concomitant tyrosine kinase inhibitor therapy (HR 1.923, 95% CI 1.077–3.434) [107]. SHAP attribution ranked tyrosine kinase inhibitor use highest (0.741) and glycyrrhizic acid class hepatoprotectants second (0.381). Almost all events were hypothyroidism: of the patients who developed thyroid dysfunction, only four were hyperthyroid.

The study reports an association rather than a mechanism. It did not measure whether liver injury fell as a consequence of treatment or whether total irAE burden changed, so it cannot show that toxicity was shifted between organs rather than reduced; the authors discuss several candidate mechanisms—glucocorticoid-like activity, the hypothalamic–pituitary–thyroid axis, deiodinase activity and immunomodulation—but the study did not test those hypotheses, and neither the HMGB1 nor the FXR/PXR framework predicts the finding. What it raises is the possibility of an organ-specific difference in irAE timing, which would require replication in an independent dataset before it is given weight. The authors report an E-value of 2.33 for the exposure–outcome association and repeated the analysis under inverse probability of treatment weighting with consistent results [107], which bounds the unmeasured-confounding objection without removing it. The weighted model table cannot be fully recomputed from the statistics reported alongside it, so the weighted analysis is not treated here as independent verification, and the other limitations remain [107]: the hazard ratio’s lower bound (1.032) lies close to unity, and confounding by indication is specifically plausible because the decision to give prophylactic hepatoprotection is itself a judgment that the patient is at elevated risk of organ injury. Thyroid dysfunction is also among the most common endocrine irAEs of PD-1 inhibitors [108,109], and whether monitoring intensity differed between groups is not reported, so detection bias cannot be excluded in either direction and the association requires prospective confirmation.

### 9.3. Evidence Pointing the Other Way: Steroid Sparing

A dose association was observed in the matched cohort (Section 7.1): among patients with high-grade liver injury (grade 3–4), 1 of 93 (1.1%) received an average daily methylprednisolone-equivalent dose of ≥1.5 mg/kg over the recorded steroid course, against 8 of 89 (9.0%) of the steroid-monotherapy group (*p* = 0.017); for the whole cohort the corresponding counts were 2 of 114 versus 9 of 115 (*p* = 0.059) [1]. The counts behind those percentages are small, so the association is hypothesis-generating rather than conclusive, and because the cohort recorded doses rather than concentrations, it shows neither lower cumulative dosing nor lower systemic exposure. Because early steroid exposure for irAEs has itself been associated with a smaller survival benefit [2], requiring less steroid is a plausible route to preserved ICI efficacy—the opposite of the theoretical concern. More speculatively, the compartment-specific HMGB1 argument of Section 4 predicts that HMGB1 blockade should enhance rather than impair checkpoint blockade, with indirect preclinical support [63]; but whether MgIG engages HMGB1 at all remains unverified (Section 8).

A pharmacokinetic caveat belongs with the dose argument. Two small studies in healthy men reported altered prednisolone pharmacokinetics with glycyrrhizin, and their designs differ in a way that changes how the finding is read: the first gave 200 mg glycyrrhizin intravenously before an intravenous dose of prednisolone hemisuccinate, and the significant changes were those of free prednisolone—the pharmacologically active fraction; the second gave oral glycyrrhizin as pretreatment and reported significant changes for total prednisolone [110,111]. In both, plasma clearance fell, the area under the curve and the mean residence time rose, and the volume of distribution was unchanged, which the authors attribute to inhibited prednisolone metabolism rather than to altered distribution. Both used glycyrrhizin itself, the natural glycoside, not MgIG; neither identifies the enzyme responsible; and neither shows that the same interaction applies to MgIG or to every corticosteroid used to treat ChILI. What they do establish is a possibility the dose categories do not by themselves exclude: if a member of this class also raises glucocorticoid exposure per milligram, a lower nominal dose need not mean lower exposure—an inference from the two prednisolone studies above [110,111], not a measured interaction with MgIG. The matched cohort reported doses, not drug concentrations [1]. A trial that proposes steroid sparing as the route by which antitumour immunity is preserved therefore has to quantify the corticosteroid actually used—its own pharmacokinetic variables, not only a methylprednisolone-equivalent dose—and record the cumulative dose given as well, which remains necessary but does not substitute for that measurement (Section 12).

### 9.4. Why Existing Data Cannot Settle the Question

The question is hard to resolve from published work: antitumour efficacy—progression-free and overall survival—was not an endpoint in either clinical dataset [1,5], so the concern has been argued rather than measured; the thyroid finding rests on a single dataset; and benefit and harm pull in opposite directions, so no single outcome can settle them. The question also turns on which preparation is meant: if the 18α aglycone corresponding to MgIG spares 11β-HSD2 up to 20 µM while the 18β aglycone inhibits it [23], toxicity signals from unstandardized licorice products cannot be attributed to MgIG, and a safety analysis that does not specify the isomer cannot support preparation-specific or isomer-specific conclusions.

### 9.5. What Would Resolve It

What would settle the question is a randomized trial with an antitumour efficacy endpoint alongside irAE ascertainment—the design specified in Section 12. In the interim, three analyses are feasible on existing data: measuring progression-free and overall survival in the two assembled cohorts [1,5], although the single-arm cohort’s follow-up ended at liver injury or after 4–6 cycles; replicating the thyroid analysis in an independent real-world dataset; and, if it replicates, testing whether the signal is isomer-dependent.

## 10. Benefit and Risk

The evidence assembled above does not support a recommendation for practice. For an ICI-treated patient who develops ChILI, the direct evidence is one propensity-score-matched retrospective case–control study [1]; for prophylaxis, the only supporting study identified here is single-arm [5]. No randomized trial in this population was identified by the search described in Section 2, and the immune-safety question is unresolved: current evidence is insufficient to determine whether adding an isomer-specified preparation alters antitumour efficacy or overall benefit–risk, and the absence of a harm signal is not evidence of safety. Table 2 sets out, question by question, the direct evidence that exists for each, its principal limitations and the conclusions it does not support.

Product fact, not an evidence rating. The licensed MgIG product has a defined 18α identity, stated in its approved chemical name and in the national reference standard for the substance [3,22]. In direct enzyme assays the 18β aglycone inhibits 11β-HSD2, which the 18α aglycone does not inhibit up to 20 µM in vitro [23]; that is an in vitro difference between aglycones, and what it means for patients given MgIG is unresolved—hypokalemia has been reported with MgIG [5,31], and a single-arm series of 1511 patients reported means only [97] (Section 11.1).

Figure 2 sets the same evidence out as two independent branches—a mechanistic-validation branch and a clinical-evidence branch—annotating each step with the strongest evidence that exists for it: the mechanistic branch is untested at every step but one, and the clinical branch is observational only for hepatoprotection. The two branches converge only on the benefit–risk question, and deliberately not as a causal sequence: if MgIG does not engage HMGB1, it could still protect the liver through the FXR/PXR–bile acid arm or another anti-inflammatory route. The figure is not a treatment algorithm: we take no position on when corticosteroid treatment for ChILI should be started, because practice for grade 2 injury is not uniform. Where corticosteroid treatment is otherwise clinically indicated, the published cohort gave MgIG 200 mg/day for a median of 8 days alongside it [1]. Potassium and blood pressure should be monitored during such use (Section 11.4 and Section 12); the cohort did not report doing so. Prophylactic use is harder to justify outside a trial, because the only supporting study identified here lacked a comparator.

The framework does not extend to two common practices. It does not support chronic self-administration of unstandardized licorice products by patients receiving ICIs, because such products are dominated by the 18β isomer and carry the mineralocorticoid risk documented for 18β glycyrrhizin preparations [28]; the 18α preparation’s own risk is expected to be different rather than absent, and hypokalemia has been reported with MgIG [5,31]. Nor does it support treating the class as interchangeable with steroids: the two differ in mechanism, in the irAE they address, and in their interaction with antitumour immunity.

## 11. Safety Considerations in Oncology Populations

### 11.1. Mineralocorticoid Effects, and Why the Isomer Story Is More Complex than It First Appears

The characteristic toxicity of licorice is pseudoaldosteronism—hypokalemia, sodium retention and hypertension—arising from inhibition of renal 11β-HSD2, which allows cortisol to activate the mineralocorticoid receptor. The 18β isomer is responsible, while MgIG is the 18α glycoside, whose aglycone leaves 11β-HSD2 uninhibited in vitro even at 20 µM [23] (Section 3.1). That is a reason to expect a different mineralocorticoid profile, not none, and the expectation has to be tested against MgIG’s own clinical record rather than against an enzyme assay.

That record is mixed, and it does not support a conclusion of safety (Table 3). Hypokalemia and blood-pressure elevation are reported with MgIG in a meta-analysis of ten studies [31] and in two retrospective series of 300 patients each, with age, daily dose and treatment duration among the associated factors [112,113]; both series are single-center and uncontrolled, so neither gives a causal incidence rate, but they make hypokalemia a clinical question rather than a theoretical one. Against that, a 1511-patient single-arm series found no significant change in mean serum potassium or blood pressure [97], a comparison of means that cannot exclude events in individuals, while in the ICI cohorts one grade 1 event occurred among 67 patients given prophylaxis [5] and the matched cohort’s laboratory panel reported no potassium, blood-pressure or aldosterone data [1]. No study identified here measures the difference between the isomers in patients, and the pharmacological argument must not be read as a safety finding.

Case reports continue to document the 18β-mediated syndrome—hypokalemia from herbal tea [25], refractory hypokalemia with hypertension [117], severe hypokalemia with uncontrolled hypertension that corrected once the licorice was stopped [26], and Japanese traditional formulations [27]—and an early series in which hypokalemia and sodium retention appeared only in diabetic patients receiving insulin [28]. These concern licorice-containing products generally, not MgIG. One case of severe hypokalemic rhabdomyolysis is reported as the first in which licorice exposure coincided with genetically confirmed Gitelman syndrome and diabetes; the authors attribute the severity to that combination [24].

The stereochemical argument is not a simple opposition between a safe 18α isomer and a toxic 18β one. A second axis has been implicated at carbon 3: 3-epi-18β-GA had a sevenfold longer elimination half-life and a 22-fold higher AUC than 18β-GA after intravenous administration in rats [118], and in 78 patients taking licorice-containing Kampo medicines its concentration, and that of its glucuronide, correlated with the diagnostic marker for pseudoaldosteronism [115]. This may explain the interindividual variability in licorice toxicity that HSD2 inhibition alone does not supply, so a compound’s isomer content at C18 may be only part of its toxicological profile.

The relationship between the C18 isomers is also not simply antagonistic: rats injured with carbon tetrachloride fared better when the two isomers were given together than with either alone, and a 4:6 ratio of 18α to 18β worked best [119]. If confirmed, this implies that both isomers contribute to hepatoprotection and that an optimal proportion exists—not what a straightforward reading of the HSD2 selectivity data would predict.

### 11.2. Who Is Actually at Risk, and at What Dose

The epidemiology is less settled than clinical intuition suggests. A review of 104 published case reports did not show enrichment for female sex or advanced age, contrary to most published statements [116]; but case-report series cannot establish or exclude demographic risk factors, because they lack an exposed-population denominator. The demographic profile of this toxicity is more often asserted than measured.

The dose–response relationship is better established than the demographic one, and more concerning than commonly assumed. A meta-analysis of randomized trials found that glycyrrhizic-acid-predominant interventions raise blood pressure, whereas flavonoid-predominant ones did not [120]. More directly, a randomized crossover trial with independently quantified glycyrrhizic acid content (Table 3) found that 100 mg/day, a dose previously regarded as unlikely to cause adverse effects, still affected renin, aldosterone and home blood pressure in healthy young volunteers [114]. The threshold for a measurable biological effect therefore lies at or below the level previously treated as safe, and well below what a patient consuming licorice confectionery, tea or a traditional formulation might ingest. This concerns unstandardized licorice, in which the 18β isomer predominates; it is not evidence about MgIG (Section 11.4).

Comorbidity and concomitant medication should be considered when assessing electrolyte risk. Potassium-wasting diuretics can aggravate hypokalemia; renin–angiotensin-system inhibitors, including ACE inhibitors and angiotensin receptor blockers, affect potassium in a different direction and require separate consideration rather than being pooled with the diuretics. This argues for explicit product specification rather than against the class (Section 11.5); the published trials have not addressed comorbidity by subgroup.

### 11.3. What the ICI Studies Reported

On the laboratory testing that cohort did report—complete blood count and renal function—MgIG plus steroid was comparable to steroid alone [1]. The single-arm prevention study recorded adverse events that were mostly grade 1–2: grade 1–2 anemia in 19 patients (28.4%) and constipation (26.9%), one further patient each with grade 3 anemia and grade 3 thrombocytopenia, and hypomagnesemia and hypermagnesemia once each (1.5%); no serious event was attributed to MgIG [5]. Neither report gives serial potassium values, and neither reports thyroid-function data—a gap given the effect of 18β-rich licorice on the renin–angiotensin–aldosterone axis at doses previously regarded as safe [114].

### 11.4. Monitoring

Pending better data, monitoring here is a precaution, not a prediction. The 18α aglycone’s failure to inhibit 11β-HSD2 in vitro does not establish that MgIG is free of the risk in patients: hypokalemia has been reported with MgIG itself, and the largest series was a single-arm comparison of means (Section 11.1). Serum potassium and blood pressure should therefore be checked before and during treatment, and the result recorded rather than assumed normal; the low threshold for measurable effects of 18β-rich licorice (Section 11.2) is a further reason to monitor, without treating that threshold as a property of MgIG [114]. Routine thyroid monitoring should continue on the schedule already used for patients receiving ICIs: the cohort described in Section 9.2 found the time to thyroid dysfunction markedly shorter under prophylactic hepatoprotection, but exposure was defined as any glycyrrhizic-acid-class hepatoprotectant, five preparations were in use with no counts and no isomer identity given, and the authors note that the appropriateness of the hepatoprotectant used was not assessed [107]. A dietary history is also warranted, since patients rarely regard licorice confectionery or tea as medication.

### 11.5. Product Specification as a Safety Issue

The most actionable safety recommendation from this review concerns product identity. The licensed 18α identity is not in doubt at the regulatory level (Section 10); what is missing is that the pharmacological literature does not carry it forward, since at least one physicochemical report assigns the opposite configuration (18β) [36] (Section 3.3). Regulatory filings and product labels should state isomer identity and purity, and the proportion where a defined mixture is used, since the one study to compare proportions found a non-obvious optimum [119].

Specification would also make the published literature interpretable: several preclinical studies of “glycyrrhizic acid” do not state which isomer, or which proportion, was used, and the C3-epimer findings above suggest that metabolite profile may matter as much as the administered isomer [115,118].

One further interaction mechanism warrants mention in an oncology context. Five *Glycyrrhiza* species, and constituents isolated from them, raised the transcriptional activity of PXR and of the aryl hydrocarbon receptor, with matching increases in cytochrome P450 3A4 (CYP3A4) and 1A2 (CYP1A2) activity [121]. Because many antineoplastic agents are CYP3A4 substrates, licorice intake could thereby alter the exposure of concurrent cancer therapy—and this is the same nuclear receptor whose upregulation has been proposed as a hepatoprotective mechanism [14,104]. The combination most worth studying is already common in practice: ICIs are increasingly given with tyrosine kinase inhibitors such as lenvatinib, whose metabolism runs through multiple pathways—CYP3A4 principally, with aldehyde oxidase contributing and biliary excretion of the metabolites [122]. The clinical consequence of dual CYP3A4 and P-glycoprotein induction can be read off directly: multiple-dose rifampicin lowered lenvatinib AUC by only 18% with C_max_ unchanged, whereas single-dose rifampicin raised AUC by 31% through P-glycoprotein inhibition [122]. Neither the magnitude nor the direction of an induction effect can therefore be inferred from in vitro CYP3A4 data alone, and a multiple-pathway substrate should be relatively protected, since interactions are most consequential when elimination proceeds mainly through a single pathway [123]. We describe this as a theoretical herb–drug interaction warranting a pharmacokinetic study, not as an anticipated reduction in efficacy—the study is worth doing because the same co-administration setting is independently associated with earlier thyroid dysfunction (HR 1.923, 95% CI 1.077–3.434) [107], and because the only human pharmacokinetic evidence identified here for a class member given with a co-administered antineoplastic agent comes from a single pilot study of 18 patients (Section 3.3) [42].

## 12. Research Agenda

The experiments below are ordered by how much they would change our understanding; they follow from the framework’s predictions in Section 8.

1. Determine whether MgIG engages HMGB1, and whether the liver effect depends on it. This experiment carries the framework, and it has two parts. First, purified 18α and 18β aglycones should be compared at matched concentrations in the same HMGB1 binding assay and the same HMGB1-dependent functional readout, with the intact glycosides included and all three redox forms of HMGB1 in the assay: a compound that prefers one form would be a lead for optimization, whereas equal engagement would mean that selectivity is not how this class works. Concentrations achievable free at the site of action are what make the comparison interpretable, because the isomers differ in absorption and tissue distribution as well as in stereochemistry. Second, the same model should measure how much of the protection depends on HMGB1, in a factorial design: MgIG or vehicle crossed with removal of the pathway by neutralization, knockdown or receptor blockade, or a sham removal, with baseline injury severity matched and the adequacy of removal verified by a target-engagement readout. The endpoint is the fraction of protection lost—a dependence estimate conditional on those assumptions, not the mechanism’s contribution, because incomplete blockade, action at more than one target, and the reduced room for protection each leave a residual that would otherwise be read as independence. Until both parts are done, the mechanism of Section 4 remains unfalsified but unanchored.

2. Characterize HMGB1 in checkpoint inhibitor hepatitis. HMGB1 participation in ChILI has not been demonstrated in any study identified here, although it predicts checkpoint inhibitor pneumonitis [53,54]. Hepatic HMGB1 release, its redox state and its relationship to injury severity in ChILI should be described before the framework is applied to the liver.

3. Test whether serum HMGB1 predicts benefit, rather than how severe the injury is. Prediction 1 (Section 8) specifies the analysis: a treatment × baseline-HMGB1 interaction with a comparator arm and pre-treatment sampling, since only the matched cohort has a comparator [1] and pre-treatment serum is not documented for either study. A correlation between HMGB1 and recovery within a single arm describes prognosis, not prediction, and the population the biomarker would be used in—ICI-treated patients who develop ChILI—must be the population it is measured in.

4. A randomized controlled trial with an efficacy endpoint. The most important unmet need is a trial that measures antitumour outcomes alongside irAE outcomes. Design considerations:

- **Design**. A randomized trial in adults with solid tumors receiving ICIs who develop ChILI for which corticosteroid treatment is clinically indicated: standard corticosteroid plus isomer-specified MgIG 200 mg/day [1], compared with standard corticosteroid plus matching placebo, with a dose-finding run-in. A treatment design is more conservative than prophylaxis, which would commit patients to treatment before injury has occurred. Blinding follows the options in Section S4: a placebo-controlled double-blind adjunctive design is preferable where feasible, and open-label treatment with blinded endpoint adjudication is acceptable where it is not.
- **Endpoints**. Primary: time to ALT normalization, or the proportion normalized at day 10, using the definitions that generated the existing effect estimate [1]; because hepatocellular and cholestatic or mixed injury are different phenotypes, ALP and total bilirubin should be reported with it, and international normalized ratio (INR) where injury is severe, with obstruction, infection, progression and concomitant drugs excluded as causes rather than assumed away. Key efficacy: progression-free survival, with overall survival secondary, both for signal detection, since a trial of this size would not be powered for them unless it were enlarged. Adequate power would require sufficient patients and outcome events; registry linkage could support follow-up but would not by itself ensure adequate power; a difference that does not reach significance would not establish preserved efficacy, and a formal claim of preservation would require a prespecified acceptable loss, an adequate sample size and a non-inferiority analysis. Safety: potassium, blood pressure, thyroid function and irAE incidence by organ, to test whether the thyroid signal [107] replicates and is isomer-dependent. Treatment context: corticosteroid starting dose, tapering and rescue rules should be protocolized, and ICI interruption or restart and any concomitant antineoplastic regimen recorded, since those decisions otherwise confound the interpretation of reduced steroid use. Pharmacokinetic: quantify the corticosteroid actually used in both arms—the agent, and its own clearance, AUC and volume of distribution, since the class has been reported to slow prednisolone elimination in small studies that used glycyrrhizin itself [110,111] and methylprednisolone equivalence does not imply equivalent AUC or equivalent interaction; the cumulative dose administered should be recorded alongside those variables, not instead of them (Section 9.3).
- **Sample size and blinding**. The only usable preliminary estimate is the matched cohort’s day 10 ALT normalization, 30.7% with MgIG plus steroid against 17.4% with steroid alone (adjusted odds ratio 2.13, 1.11–4.09) [1]; because observational estimates are generally optimistic, a confirmatory trial should be powered for a smaller difference, and the 9.0% prophylaxis incidence [5] cannot anchor the calculation. Isomer identity and purity must be prespecified as product quality attributes; A trial in this population is not large by oncology standards. With 1:1 allocation, a two-sided alpha of 0.05 and 80% power, detecting an increase in day-10 ALT normalization from 17.4% with corticosteroid alone [1] to 25% would require about 451 evaluable patients per arm—497 per arm allowing for 10% dropout, 994 in total. Powering for the full observed difference (30.7%) would roughly halve that requirement. Follow-up is short for the primary endpoint (day 10), with safety follow-up to day 28 and survival follow-up for 12 months for the secondary endpoints.

5. Replicate or refute the thyroid signal. If it replicates, test whether it is isomer-dependent, and measure irAE incidence across organs rather than the thyroid alone, so that a genuine trade-off can be distinguished from an isolated thyroid effect.

6. Extend the evidence beyond the liver. Whether the class modifies the incidence or severity of ICI-related colitis, pneumonitis or myocarditis is unknown; whether protection tracks organ by organ is testable in existing irAE registries, though HMGB1 dependence would still need a mechanistic study in each organ.

7. Determine whether the class modulates checkpoint molecules in humans. No human study identified here isolates the effect of a glycyrrhizic-acid-class compound on PD-1 or PD-L1 expression: the autoimmune-hepatitis comparison measured these outcomes, but both arms received MgIG and differed in the added immunosuppressant [43]. The preclinical work reporting direct molecular modulation of PD-L1 concerns flavonoids rather than triterpenoids [72,73,74,75,77], whereas the triterpenoid studies report checkpoint-phenotype changes in immune cells as part of a remodeled microenvironment—a different kind of evidence, which a pharmacodynamic study in ICI-treated patients could separate.

## 13. Conclusions

The glycyrrhizic acid class occupies an unusual position in ICI supportive care: it is already used in practice, it has a plausible mechanism, and it now has a multicentre propensity-score-matched clinical dataset in checkpoint inhibitor hepatitis—yet no review identified here assembles that link while also asking whether protecting the liver preserves or compromises checkpoint blockade. Three conclusions follow.

First, the class is not a single entity, and its isomers differ in both target and toxicity: the 18β isomer carries the mineralocorticoid toxicity recognized for decades, whereas the 18α aglycone corresponding to MgIG does not inhibit 11β-HSD2 up to 20 µM in vitro. That argues for a different mineralocorticoid risk, not a lower one that can be assumed, and no study identified here has compared the isomers on a mineralocorticoid endpoint in patients (Section 11.1) [23]. Studies that treat “glycyrrhizic acid” as one substance, including several analyzed here, do not state the isomer or preparation used, which limits extrapolation across isomers and formulations.

Second, a clinically relevant signal has emerged in checkpoint inhibitor hepatitis: in a multicentre propensity-score-matched cohort, adding MgIG to steroids was associated with faster biochemical recovery, a lower proportion of patients reaching the average daily corticosteroid dose threshold in the grade 3–4 subgroup and shorter hospital stay [1], and a single-arm prevention study reported study-defined liver injury in 9.0% against a threshold reported as prespecified [5]. Both datasets concern the liver alone; neither was randomized, and neither reported antitumour efficacy.

Third, the mechanistic framework remains a hypothesis whose two premises are unverified (Section 8), and its preclinical support is indirect [50,63,65,66]. Because HMGB1’s extracellular activity is redox-state dependent and the protein also supports antitumour immune priming in some settings, blockade is not predictably beneficial on either side of the model, and whether any member of this class discriminates between the forms is untested (Section 5.1) [20,21,47,48].

This review does not recommend the class for ChILI outside a trial and proposes no treatment algorithm: the dose published in the cohort and the potassium and blood-pressure checks are set out in Section 11.4 and Section 12 with their provenance. Where corticosteroid treatment is otherwise indicated, monitoring of potassium, blood pressure and—pending clarification—thyroid function remains necessary, and prophylaxis outside a trial is not supported. The question that would most change practice, whether hepatoprotection with this class preserves or compromises the benefit of checkpoint inhibition, has not yet been asked in a design capable of answering it.

## Figures and Tables

**Table 1:** Clinical studies of glycyrrhizic acid class compounds, in patients receiving antineoplastic therapy and in chronic liver disease.

Study	Design and Population	Intervention	Comparator	Key Outcomes
Zhi et al., 2025 [1]	Multicentre, retrospective, propensity-score-matched case–control; checkpoint inhibitor-induced liver injury	MgIG 200 mg/day (median 8 days) plus corticosteroid; n = 114	Corticosteroid alone; n = 115	ALT reduction −148 vs. −98 U/L (*p* = 0.010); adjusted odds ratio for ALT normalization 2.13 (1.11–4.09); among patients with grade 3–4 injury, an average daily methylprednisolone-equivalent dose of ≥1.5 mg/kg over the recorded steroid course in 1 of 93 (1.1%) vs. 8 of 89 (9.0%) (*p* = 0.017); corresponding counts for the whole cohort 2 of 114 vs. 9 of 115 (*p* = 0.059)
Guo et al., 2026 [5]	Single-arm, prospective; advanced cancer starting ICI-based regimens	Prophylactic MgIG 200 mg/day, planned 4–6 cycles (53 of 67 completed 4–6); n = 67	None	Study-defined liver injury 9.0% (6/67; 95% CI 3.4–18.5%) against a threshold reported as prespecified (failure declared if the confidence-interval upper limit exceeded 20%); grade I–II 83.3% (5/6), one grade III ALT elevation, no grade IV
Qin et al., 2017 (MAGIC-301) [95]	Prospective, open-label, randomized, 28 centers; 1200 enrolled, 1146 in the full analysis set; chemotherapy of defined hepatotoxic risk	MgIG 200 mg/day from the day before chemotherapy, protocol target ≥7 days, minimum 5; n = 766	Chemotherapy alone; n = 380	Liver injury 52.81% vs. 62.63% (*p* = 0.0004 as reported; recomputation from the published counts gives *p* ≈ 0.002, Section S3); overall NCI grade distribution shifted in favor of MgIG (Wilcoxon *p* = 0.0008) while the grade ≥2 rate itself, 10.07% vs. 12.37%, did not differ significantly; ALT abnormality at the day 13–15 visit 8.63% vs. 16.58%, AST 10.72% vs. 20.05% (both *p* < 0.0001); rescue treatment 1.3% vs. 9.2% (*p* < 0.0001)
Yan et al., 2015 [96]	Prospective, randomized; treatment-naive gastrointestinal malignancies receiving FOLFOX or XELOX	Prophylactic MgIG; n = 114	Glutathione; n = 102	Abnormal liver biochemistry in 12 vs. 28 chemotherapy courses (*p* < 0.01); no grade II or higher hepatotoxicity in either arm
Li et al., 2019 [97]	Single-arm before-after, no control group; hematologic malignancy	MgIG 200 mg/day, ≥7 days; n = 1511	None	Liver biochemical indices not increased from baseline; most common abnormality total bilirubin, 15.19%
Hu et al., 2023 [31]	Meta-analysis of 10 studies; chronic liver disease with elevated ALT	MgIG 200 vs. 100 mg/day; n = 1522	100 mg/day	Greater reduction of ALT (MD −30.73, 95% CI −52.52 to −8.94), AST (MD −34.30) and total bilirubin (MD −15.37) at 200 mg/day; no difference in adverse events
Li et al., 2021 [43]	Randomized, single-center; autoimmune hepatitis	Oral MgIG plus tripterygium glycosides, 24 weeks; n = 38	Oral MgIG alone; n = 38	Lower peripheral PD-L1^+^/CD4^+^, PD-L1^+^/CD8^+^, PD-1^+^/CD4^+^ and PD-1^+^/CD8^+^ percentages and better short-term biochemical response (attribution discussed in Section 7.3)

ALT, alanine aminotransferase; AST, aspartate aminotransferase; CI, confidence interval; ICI, immune checkpoint inhibitor; MD, mean difference; MgIG, magnesium isoglycyrrhizinate.

**Table 2:** Benefit–risk assessment of glycyrrhizic acid class hepatoprotection in ICI-treated patients, by question: the direct evidence that exists, its principal limitations, and what that evidence does not support.

Question	Direct Evidence Available	Principal Limitations	Does Not Support
Does it accelerate biochemical recovery in ChILI?	One propensity-score-matched retrospective case–control study: greater ALT, AST, ALP and bilirubin reduction, higher ALT normalization at day 10, and ALP and bilirubin normalization higher only at discharge [1]	Retrospective, 114 versus 115 patients; matching balances measured confounders but cannot exclude confounding by indication; the source itself calls for prospective confirmation	That the association is causal, that it generalizes beyond this cohort, or that endpoints other than the reported biochemical ones behave the same way
Was recorded corticosteroid dosing lower?	The same cohort: a lower proportion reaching the average daily threshold of ≥1.5 mg/kg methylprednisolone-equivalent over the recorded course in the grade 3–4 subgroup, 1 of 93 versus 8 of 89; whole-cohort counts 2 of 114 versus 9 of 115, not significant [1]	A secondary endpoint; a small subgroup; doses were recorded, not plasma concentrations; starting dose, tapering and rescue were clinician decisions	That cumulative dosing or systemic glucocorticoid exposure was lower, or that the class has a causal steroid-sparing effect (Section 9.3)
Does prophylaxis prevent ChILI?	One prospective single-arm study: study-defined liver injury in 6 of 67 patients (9.0%, 95% CI 3.4–18.5%) against a failure threshold reported as prespecified (upper confidence limit above 20%) [5]	No concurrent control, so the comparison is historical; every patient received an ICI-containing combination regimen and 40.3% had corticosteroid premedication; 53 of 67 completed 4–6 cycles, and the per-protocol subset excluded patients who had already developed injury	That the incidence would be lower than without prophylaxis, or that 9.0% is an adjudicated immune-hepatitis rate
Does it protect organs other than the liver?	No direct clinical evidence identified here; the clinical datasets concern the liver alone	Kidney, heart and lung findings are preclinical, and use different compounds and models	That protection extends to other irAE-affected organs in patients
Does it alter the irAE profile?	One single-center observational cohort: earlier thyroid dysfunction with pooled glycyrrhizic acid class hepatoprotectants, adjusted HR 1.774 (95% CI 1.032–3.051) [107]	Observational; five preparations pooled, with no formulation-specific estimate; hazard-ratio lower bound close to the null; monitoring intensity not described; neither liver injury nor total irAE burden was reported	That the signal belongs to MgIG or to the 18α isomer, that toxicity is redistributed between organs, or that survival is affected
Does it preserve or impair ICI efficacy?	No antitumour endpoint was reported in either clinical dataset [1,5]	Progression-free and overall survival were not among the reported outcomes, and neither study was designed or powered for them	Any statement about antitumour efficacy or its preservation; the absence of a harm signal is not evidence of safety
What dose and duration?	200 mg/day for a median of 8 days alongside corticosteroid in the matched cohort [1]; 200 mg for 4–6 cycles prophylactically [5]	The only dose comparison is in chronic liver disease, not in an oncology population [31]; no dose-finding study in ICI-treated patients	That 200 mg/day is optimal, or that another dose or duration would not be better

**Table 3:** Safety-relevant clinical evidence for glycyrrhizic acid class compounds and licorice intake.

Study	Design and Population	Exposure	Comparator	Key Outcome
Li et al., 2026 [107]	Observational (real-world) cohort; patients starting PD-1 inhibitors with normal baseline thyroid function	Prophylactic glycyrrhizic acid class hepatoprotectants, five preparations pooled; n = 62	No prophylaxis; n = 113	Thyroid dysfunction HR 1.774 (95% CI 1.032–3.051); unweighted Kaplan–Meier time to 50% cumulative incidence of thyroid dysfunction 2.5 vs. 8 months (*p* < 0.001); E-value 2.33
Zhi et al., 2025 [1]	Multicentre propensity-score-matched case–control (matched 1:1 on sex, age and initial ALT at injury onset; cancer type, comorbidity and ICI type not matched); checkpoint inhibitor-induced liver injury	MgIG plus corticosteroid; n = 114	Corticosteroid alone; n = 115	Laboratory safety profile comparable to steroid monotherapy; reported no potassium, blood-pressure, aldosterone or thyroid-function data
Hu et al., 2023 [31]	Meta-analysis of 10 studies; chronic liver disease with elevated ALT	MgIG 200 or 100 mg/day; n = 1522	100 mg/day	Hypokalemia and blood-pressure elevation among the adverse reactions reported across the included studies; no significant difference in adverse-event rates between the two doses
Li et al., 2019 [97]	Single-arm before-after, no control group; hematologic malignancy	MgIG 200 mg/day, ≥7 days; n = 1511	None	Mean serum potassium 4.54 → 4.41 mmol/L (*p* = 0.518) and mean blood pressure unchanged across the treatment course; means only, from a single arm, so individual events cannot be excluded
Huang et al., 2024 [112]	Retrospective series; inpatients treated with MgIG injection	MgIG injection; n = 300	None	Hypokalemia in 74 (24.67%); sex, age and concomitant antibacterials independently associated, and a risk score published; single-center and uncontrolled, so not a causal incidence rate
Wang et al., 2023 [113]	Retrospective series; patients treated with MgIG injection	MgIG injection; n = 300	None	Hypertension in 41 (13.67%) and hypokalemia in 29 (9.67%); older age, higher body mass index and longer course associated with hypertension; older age, higher daily dose and longer course with hypokalemia
Af Geijerstam et al., 2024 [114]	Randomized, controlled, crossover; healthy volunteers, median age 24 years	Licorice containing 100 mg glycyrrhizic acid daily for 2 weeks; n = 28	Control product	Significant effects on renin, aldosterone and home blood pressure at a dose previously regarded as unlikely to cause adverse effects
Sakoda et al., 2024 [115]	Clinical pharmacokinetic correlation; patients taking licorice-containing Kampo medicines	Licorice-containing formulations; n = 78	—	Concentrations of 3-epi-18β-GA and its glucuronide correlated with the diagnostic marker for pseudoaldosteronism
Ceccuzzi et al., 2023 [116]	Narrative review of published case reports	Licorice overconsumption; 104 cases	—	No enrichment for female sex or advanced age among the reported cases; a case series cannot establish or exclude demographic risk factors

## Data Availability

This review analyzed only previously published literature; no new datasets were generated. The search strategies, the reference library used to format citations, and the source code for the two figures are available from the corresponding author on reasonable request.

## Supplementary Materials

The following supporting information can be downloaded at: https://www.mdpi.com/article/10.3390/ijms27219987/s1.

## Abbreviations

The following abbreviations are used in this manuscript:

ALP	alkaline phosphatase
ALT	alanine aminotransferase
AST	aspartate aminotransferase
AUC	area under the concentration–time curve
ChILI	checkpoint inhibitor-induced liver injury
CI	confidence interval
CK-MB	creatine kinase-MB
ConA	concanavalin A
FXR	farnesoid X receptor
GA	glycyrrhetinic acid
GSDMD	gasdermin D
HMGB1	high-mobility group box 1
HR	hazard ratio
HSD11B1	11β-hydroxysteroid dehydrogenase type 1
HSP90	heat shock protein 90
ICI	immune checkpoint inhibitor
ICIs	immune checkpoint inhibitors
irAE	immune-related adverse event
MAPK	mitogen-activated protein kinase
MDSC	myeloid-derived suppressor cell
MgIG	magnesium isoglycyrrhizinate
PD-L1	programmed death-ligand 1
PXR	pregnane X receptor
RAGE	receptor for advanced glycation end-products
TLR2	Toll-like receptor 2
TLR2/TLR4	Toll-like receptors 2 and 4
TLRs	Toll-like receptors
Treg	regulatory T-cell
C_max_	maximum plasma concentration
INR	international normalized ratio
CYP3A4	cytochrome P450 3A4
CYP1A2	cytochrome P450 1A2
PRISMA	Preferred Reporting Items for Systematic Reviews and Meta-Analyses
MEDLINE	Medical Literature Analysis and Retrieval System Online
PMC	PubMed Central
DOAJ	Directory of Open Access Journals
CNKI	China National Knowledge Infrastructure
