# Supplementary material for: Glycyrrhizic Acid Class Compounds in Immune Checkpoint Inhibitor Therapy: Stereochemistry, Mechanistic Hypotheses, and Protection Against Immune-Related Organ Injury
Source: Int J Mol Sci. 2026 Nov 7;27(21):9987. doi: 10.3390/ijms27219987 (PMC13763845; doi:10.3390/ijms27219987)
Supplement: Supplementary file 1 [file ijms-27-09987-s001.zip › ijms-4660125-supplementary.pdf]

International Journal of
Molecular Sciences

MDPI

Supplementary Material

Title: Glycyrrhizic acid class compounds in immune checkpoint inhibitor therapy: stereochemistry, mechanistic hypotheses, and protection against immune-related organ injury

Table S1. Outcomes of the propensity-score-matched multicentre cohort of checkpoint inhibitor-induced liver injury, each row carrying its own time point [1]

Endpoint (day 10 unless stated)	MgIG + steroid (n = 114)	Steroid alone (n = 115)	p
Median ALT reduction, U/L	−148 (−232 to −44)	−98 (−239 to 8)	0.010
Median AST reduction, U/L	−111 (−293 to −45)	−84 (−345 to 7)	0.029
Median ALP reduction, U/L	−27 (−72 to −7)	−10 (−47 to 24)	0.002
Median total bilirubin reduction, μmol/L	−5.7 (−15.4 to −2.3)	−0.3 (−11.6 to 7.5)	<0.001
ALT normalisation	30.7%	17.4%	0.018
ALT normalisation, adjusted OR	2.13 (1.11–4.09)	reference	0.023
Average daily methylprednisolone-equivalent dose ≥1.5 mg/kg over the recorded steroid course (cumulative dose ÷ steroid days ÷ body weight); high-grade (CTCAE 3–4) subgroup, n = 93 vs 89	1 of 93 (1.1%)	8 of 89 (9.0%)	0.017
Total length of stay, days (hospitalisation endpoint, not a day-10 outcome)	15 (10–24)	17 (13–28)	0.020

Values are medians with interquartile ranges unless otherwise stated. The dose row is a subgroup analysis among patients with high-grade injury (CTCAE grade 3–4), so its percentages are not proportions of the cohort denominators given in the column heads: the corresponding whole-cohort counts were 2 of 114 versus 9 of 115 ($p = 0.059$). The dose was calculated as the cumulative dose over the recorded corticosteroid course divided by the number of steroid days and by body weight, with doses converted to methylprednisolone equivalents; it is not a maximum single-day dose, not a starting dose and not a measure of systemic exposure. This table expands the "Zhi 2025" row of Table 1 in the main text. ALT, alanine aminotransferase; ALP, alkaline phosphatase; AST, aspartate aminotransferase; MgIG, magnesium isoglycyrrhizinate; OR, odds ratio.

Table S2. Preclinical evidence for glycyrrhizic acid class compounds and licorice constituents, by compound class.

Compound	Isomer stated in source?	Model	Administration	Key finding	Ref
**A. Glycyrrhizic acid class — triterpenoids: immunostimulation and immunogenic cell death**					
Glycyrrhizic acid	**No**	Melanoma, mice	intradermal, 45 mg/kg/day, 5 alternate days	Restricts tumour progression; Th2→Th1 shift; reduces regulatory T cells and MDSCs	[68]
GA–chlorambucil prodrug	**No**	Hepatocellular carcinoma	tail-vein i.v.	Increases $CD4^+$ and $CD8^+$ T cells at the tumour site; promotes Th1 differentiation	[69]
GA	**No**	Hepatocellular carcinoma, in vitro and in vivo	intraperitoneal 20–60 mg/kg (in vivo)	Elicits immunogenic cell death via endoplasmic reticulum stress–pyroptosis cross-talk	[70]
**B. Glycyrrhizic acid class — delivery platforms**					
Glycyrrhizic acid (ferroptosis plus HMGB1 blockade)	**No**	4T1 mammary carcinoma, mice	intravenous (dose not stated)	Nanoplatform combining ferroptosis induction with HMGB1 blockade to prevent MDSC recruitment	[86]
Glycyrrhizic acid-based nanotheranostic	**No**	Hepatocellular carcinoma	tail-vein i.v., GL 2.8 mg/kg + IR780 0.6 mg/kg every other day	Suppresses HSP90-mediated thermoresistance; enhances photothermal immunotherapy	[87]
Glycyrrhizin–doxorubicin alginate nanogel	**No**	Hepatocellular carcinoma	tail-vein i.v., doxorubicin 4 mg/kg equivalent	Attenuates macrophage activation while delivering the cytotoxic agent	[88]
GA nanoparticles with ferrotherapy	**No**	Leukaemia and colorectal tumour models	tail-vein i.v., 3×/week for 2 weeks, with ferumoxytol and anti-PD-L1	Immunogenic-cell-death nanoinducer; improves checkpoint-based immunotherapy	[89]
Metformin–GA hydrogel	**Yes** — 18β stated explicitly	Murine melanoma (B16-F10)	intratumoral (in situ gelation)	Spatiotemporal microenvironment remodelling, photothermal-triggered	[91]
**C. Glycyrrhizic acid class — HMGB1-directed binding and organ protection**					

Glycyrrhizin	**No**	Purified HMGB1	in vitro	Direct binding with **Kd ≈ 150 μM**	[50]
GA derivatives (synthetic)	**No**	LPS-stimulated macrophages	in vitro	Surface-plasmon-resonance-verified HMGB1 binding, best Kd 12.5 μM	[51]
Glycyrrhizin	**No**	Experimental sepsis	intraperitoneal 50 mg/kg	Competitively blocks the HMGB1–LPS interaction and caspase-11 activation	[52]
Glycyrrhizin	**No**	Contrast-induced acute kidney injury, rats and mice	intragastric 150 mg/kg before iohexol	Inhibits HMGB1-mediated ferroptosis in renal tubular epithelial cells	[59]
Glycyrrhizic acid	**No**	Streptozotocin-induced diabetic rats	intragastric, 150 mg/kg/day for 8 weeks	Attenuates kidney injury as an HMGB1 inhibitor	[60]
Glycyrrhizin	**No**	Rabbit cardiac arrest	intravenous 4 mg/kg, 5 min after ROSC	Blunts the rises in HMGB1, IL-6 and IL-10; reduces cerebral T-cell infiltration	[62]
**D. Glycyrrhizic acid class — T-cell-mediated liver-injury models**					
MgIG (18*α*)	**Yes** — 18*α* stated explicitly	ConA 20 mg/kg intravenously, mice	intraperitoneal 30 mg/kg, 1 h before ConA	Reduces lethality, injury, cytokine levels and hepatocyte death; inhibits autophagy	[46]
MgIG (18*α*)	**Yes** — 18*α* stated explicitly	ConA mice; LPS-stimulated macrophages	intraperitoneal 5, 10, 20, 40 and 80 mg/kg, 30 min before ConA (20 mg/kg optimal)	Modulates p38 and JNK MAPK signalling	[19]
MgIG (18*α*)	**Yes** — 18*α* stated explicitly	ConA mice (autoimmune hepatitis)	intraperitoneal 50 or 100 mg/kg, 2 h before Con A	Inhibits proliferation of the $CD4^+CD25^-CD69^+$ subset	[44]
Glycyrrhizic acid	**No**	ConA mice	intraperitoneal 200 mg/kg, 1 h before ConA	Increases anti-inflammatory MoMF-1 and decreases the pro-inflammatory $iNOS^{hi}$ subset (CyTOF and single-cell RNA sequencing)	[98]
Glycyrrhizin	**No**	ConA mice, 8 weeks	intraperitoneal 10–50 mg/kg, three times weekly	Regulates the $CD4^+$ T-cell response via JNK, ERK and PI3K/AKT	[99]

Diammonium glycyrrhizinate	**Yes** — 18α stated explicitly	ConA mice	intraperitoneal 75 and 200 mg/kg, 2 h before ConA	Inhibits natural killer T-cell proliferation; promotes regulatory T cells	[100]
MgIG (18α)	**No**	ConA mice	not stated	Upregulates hepatic FXR and PXR (conference abstract)	[14]
MgIG (18α)	**Yes** — stated as a high-purity single 18-α isomer	ConA mice	intraperitoneal 12.5, 25 or 50 mg/kg, 5 consecutive days before ConA and once 1 h after	Lowers hepatic malondialdehyde and neopterin; raises superoxide dismutase; reduces myeloperoxidase	[18]
MgIG (18α)	**No**	ConA mice	not stated	Amelioration via the MAPK pathway (conference abstract)	[17]
MgIG (18α)	**No**	D-galactosamine with Freund's complete adjuvant, mice, 13 weeks	not stated	Dose-dependent improvement in liver function, reduced plasma nitric oxide and improved survival (abstract only)	[16]
GA	**No**	α-naphthyl isothiocyanate-induced cholestatic injury, mice	ANIT intragastric; GA intraperitoneal 25 or 50 mg/kg daily for 10 days	Inhibits acetylated HMGB1/TLR2-mediated crosstalk between hepatocytes and macrophages	[102]
Glycyrrhizin	**No**	Hepatic ischaemia–reperfusion injury	intravenous (tail vein) 50 mg/kg, twice	Suppresses HMGB1-dependent, GSDMD-mediated pyroptosis of Kupffer cells	[103]
**18β-GA**	**Yes** — 18β stated explicitly	D-galactosamine/LPS acute liver injury	intraperitoneal 60 mg/kg for 5 days	PXR-mediated inhibition of autophagy degradation	[104]
Glycyrrhizin	**No**	ConA hepatitis with renal involvement, mice	intraperitoneal 50 mg/kg, 1 h before Con A	Attenuates renal inflammation via the IL-25/M2 axis	[101]
**E. Adjacent constituents of *Glycyrrhiza* (not members of this class)**					
Licochalcone A	n/a (chalcone)	Colon cancer cells	in vitro; xenograft gavage 15 or 50	Inhibits proliferation and promotes apoptosis by	[72]

		(HCT116) and xenograft	mg/kg every 3 days for 30 days	targeting PD-L1 via NF-κB and Ras/Raf/MEK	
Licochalcone A	n/a (chalcone)	Lung cancer cells	in vitro	Suppresses interferon-γ-induced PD-L1 expression	[73]
Isoliquiritigenin	n/a (chalcone)	Melanoma, in vitro and in vivo	50 or 100 mg/kg/day	Represses PD-L1 via inhibition of STAT3 phosphorylation	[74]
Isoliquiritigenin	n/a (chalcone)	Breast tumour, immunocompetent versus immunocompromised mice	oral, 50 mg/kg/day	Greater efficacy in immunocompetent mice; $CD8^+$ T-cell infiltration; reduced PD-L1	[75]
G. uralensis phenolics (10 flavonoids, 4 coumarins, 2 benzofurans)	n/a (mixed phenolics)	PD-1/PD-L1 binding assay	in vitro	Weak PD-1/PD-L1 inhibition, 30–65% at 100 μM	[77]
Licorice extract (*G. uralensis*)	not applicable (extract)	Non-small cell lung cancer, in vitro and in vivo	oral gavage 200 mg/kg/day from day 2	Downregulates the CDK4–cyclin D1 complex; increases $CD8^+$ T-cell infiltration	[83]
Glycyrrhiza polysaccharides	not applicable (polysaccharide)	Mouse tumour model	oral	Increases IFN-γ-producing intestinal γδT cells via gut microbiota and TLR/NF-κB	[79]
Licoricidin	not applicable (isoflavonoid)	Cervical cancer cells	in vitro	Induces immunogenic cell death with HMGB1 and ATP release	[71]
Glycyrrhiza polysaccharide-adjuvanted liposome	not applicable (polysaccharide)	Murine melanoma	intradermal (tail base), 50 or 200 μg with 20 μg OVA, days 0/7/14	Lymph-node-targeted modulation of the dendritic cell–T-cell axis	[80]
Glabridin–NHC-Au(I) complex	not applicable (isoflavonoid)	Liver cancer models	intraperitoneal 5 mg/kg every two days	Targets TrxR and MAPK; enhances dendritic cell maturation; reduces MDSCs, M2 macrophages and regulatory T cells; suppresses PD-L1	[82]
**F. Reference comparator (not a *Glycyrrhiza* constituent)**					
**Anti-HMGB1 neutralising antibody** (not a	not applicable (antibody)	Mice, radiotherapy plus	anti-HMGB1 antibody 20 μg intraperitoneal on day 15	Reduced cardiac injury; **no detected reduction in short-term tumour control** (day-21 endpoints, n = 5 per group)	[55]

member of this class)	PD-1 inhibitor

Panels A to D list only members of the glycyrrhizic acid class. Panel E lists constituents of Glycyrrhiza that are not members of that class and is included as adjacent evidence only; panel F is a reference antibody used to probe the mechanism, not a Glycyrrhiza constituent. The isomer column records whether the source states the isomeric identity of the material it studied. The question applies to the 28 entries for which a C18 configuration is meaningful — the class compounds of panels A to D; the other 11 entries (panel E's adjacent constituents and panel F's antibody) are recorded as not applicable and are excluded from the denominator. Of those 28, 7 state the isomer: 18α in [18], [44], [19], [100] and [46]; 18β in [104] and [91]. Where a compound is named without an isomer — glycyrrhizin, glycyrrhizic acid, GA — the natural product is the 18β form, but the column still records it as unstated unless the source says so. A passing chemical description of the natural product does not count as specifying the material used. Where a row names MgIG as the 18α preparation although the source states no isomer, that identification rests on the approved product information and the national reference standard [3,22], not on the report itself. Administration is recorded where the source states it; "not stated" means it was not reported in the abstract or full text, not that the study omitted it. ConA, concanavalin A; CyTOF, cytometry by time of flight; FXR, farnesoid X receptor; GA, glycyrrhetinic acid; HMGB1, high-mobility group box 1; LPS, lipopolysaccharide; MAPK, mitogen-activated protein kinase; MDSC, myeloid-derived suppressor cell; MgIG, magnesium isoglycyrrhizinate; MoMF, monocyte-derived macrophage; PXR, pregnane X receptor; TLR, Toll-like receptor; TrxR, thioredoxin reductase.

Section S1. Search strategy

Searches were executed on 24 September 2026 with no language restriction and no lower date limit. The concept-block searches below were run in PubMed/MEDLINE (NCBI E-utilities), with all terms in the title and abstract fields; each string is given verbatim, and each was executed with the cut-off applied as a publication-date limiter — `AND ("1900/01/01"[dp] : "2026/09/24"[dp])` — so the clause below must be added to a printed string to reproduce its count exactly. Because that window spans the indexed years, the limiter removes only records whose publication date falls after the search date — repeating the searches on 12 October 2026 with and without it returned 13,047 and 13,077 for the substance block — and the counts drift upward as indexing continues, so a later re-run is slightly above the table: on that date the seven counts were 13,047, 112,850, 346,376, 24, 680, 1 and 5, the last three unchanged from the cutoff. Europe PMC, PubMed Central, Crossref, OpenAlex and DOAJ were queried separately, because their field syntax and indexing differ: they were used to identify records, to obtain full texts and to verify bibliographic and open-access status. No count from them is reported, and every number in the table below is a PubMed/MEDLINE count — none is a total across databases.

#	Query	PubMed/MEDLINE records at cutoff
S	Substance block alone	13036
C	Clinical-context block alone	112519
M	Mechanism and organ block alone	345801
S+C	Substance AND clinical context	23

S+L	Substance AND liver or irAE terms	679
S+C+L	Substance AND clinical context AND liver or irAE terms	1
S+C+H	Substance AND clinical context AND HMGB1	5

Complete Boolean strings

S — Substance block alone

("Glycyrrhiza"[tiab] OR "licorice"[tiab] OR "liquorice"[tiab] OR "glycyrrhiz"[tiab] OR "glycyrrhizin"[tiab] OR "glycyrrhizic acid"[tiab] OR "glycyrrhetinic acid"[tiab] OR "enoxolone"[tiab] OR "carbenoxolone"[tiab] OR "magnesium isoglycyrrhizinate"[tiab] OR "isoglycyrrhizinate"[tiab] OR "glycyrrhizinate"[tiab] OR "diammonium glycyrrhizinate"[tiab] OR "monoammonium glycyrrhizinate"[tiab] OR "ammonium glycyrrhizinate"[tiab] OR "18alpha-glycyrrhetinic acid"[tiab] OR "18beta-glycyrrhetinic acid"[tiab])

C — Clinical-context block alone

("immune checkpoint inhibitor"[tiab] OR "checkpoint inhibitor"[tiab] OR "checkpoint blockade"[tiab] OR "PD-1"[tiab] OR "PD-L1"[tiab] OR "CTLA-4"[tiab] OR "ipilimumab"[tiab] OR "nivolumab"[tiab] OR "pembrolizumab"[tiab] OR "atezolizumab"[tiab] OR "durvalumab"[tiab] OR "cemiplimab"[tiab] OR "avelumab"[tiab] OR "tremelimumab"[tiab] OR "dostarlimab"[tiab] OR "toripalimab"[tiab] OR "sintilimab"[tiab] OR "camrelizumab"[tiab] OR "tislelizumab"[tiab] OR "immune-related adverse event"[tiab] OR "immune-related adverse events"[tiab] OR "immune-related hepatitis"[tiab] OR "checkpoint inhibitor hepatitis"[tiab])

M — Mechanism and organ block alone

("HMGB1"[tiab] OR "high mobility group box 1"[tiab] OR "RAGE"[tiab] OR "TLR2"[tiab] OR "TLR4"[tiab] OR "NLRP3"[tiab] OR "inflammasome"[tiab] OR "caspase-1"[tiab] OR "GSDMD"[tiab] OR "pyroptosis"[tiab] OR "drug-induced liver injury"[tiab] OR "hepatotoxicity"[tiab] OR "liver injury"[tiab] OR "hepatoprotection"[tiab] OR "myocarditis"[tiab] OR "thyroiditis"[tiab] OR "hypophysitis"[tiab] OR "colitis"[tiab] OR "11beta-hydroxysteroid dehydrogenase"[tiab] OR "mineralocorticoid"[tiab] OR "pseudoaldosteronism"[tiab] OR "farnesoid X receptor"[tiab] OR "pregnane X receptor"[tiab])

S+C — Substance AND clinical context

("Glycyrrhiza"[tiab] OR "licorice"[tiab] OR "liquorice"[tiab] OR "glycyrrhiz"[tiab] OR "glycyrrhizin"[tiab] OR "glycyrrhizic acid"[tiab] OR "glycyrrhetinic acid"[tiab] OR "enoxolone"[tiab] OR "carbenoxolone"[tiab] OR "magnesium isoglycyrrhizinate"[tiab] OR "isoglycyrrhizinate"[tiab] OR "glycyrrhizinate"[tiab] OR "diammonium glycyrrhizinate"[tiab] OR "monoammonium glycyrrhizinate"[tiab] OR "ammonium glycyrrhizinate"[tiab] OR "18alpha-glycyrrhetinic acid"[tiab] OR "18beta-glycyrrhetinic acid"[tiab]) AND ("immune checkpoint inhibitor"[tiab] OR "checkpoint inhibitor"[tiab] OR "checkpoint blockade"[tiab] OR "PD-1"[tiab] OR "PD-L1"[tiab] OR "CTLA-4"[tiab] OR "ipilimumab"[tiab] OR "nivolumab"[tiab] OR "pembrolizumab"[tiab] OR "atezolizumab"[tiab] OR "durvalumab"[tiab] OR "cemiplimab"[tiab] OR "avelumab"[tiab] OR "tremelimumab"[tiab] OR "dostarlimab"[tiab] OR "toripalimab"[tiab] OR "sintilimab"[tiab] OR "camrelizumab"[tiab] OR "tislelizumab"[tiab] OR "immune-related

adverse event"[tiab] OR "immune-related adverse events"[tiab] OR "immune-related hepatitis"[tiab] OR "checkpoint inhibitor hepatitis"[tiab])

S+L — Substance AND liver or irAE terms

("Glycyrrhiza"[tiab] OR "licorice"[tiab] OR "liquorice"[tiab] OR "glycyrrhiz"[tiab] OR "glycyrrhizin"[tiab] OR "glycyrrhizic acid"[tiab] OR "glycyrrhetinic acid"[tiab] OR "enoxolone"[tiab] OR "carbenoxolone"[tiab] OR "magnesium isoglycyrrhizinate"[tiab] OR "isoglycyrrhizinate"[tiab] OR "glycyrrhizinate"[tiab] OR "diammonium glycyrrhizinate"[tiab] OR "monoammonium glycyrrhizinate"[tiab] OR "ammonium glycyrrhizinate"[tiab] OR "18alpha-glycyrrhetinic acid"[tiab] OR "18beta-glycyrrhetinic acid"[tiab]) AND ("drug-induced liver injury"[tiab] OR hepatotoxicity[tiab] OR "liver injury"[tiab] OR hepatitis[tiab] OR "immune-related adverse event"[tiab])

S+C+L — Substance AND clinical context AND liver or irAE terms

("Glycyrrhiza"[tiab] OR "licorice"[tiab] OR "liquorice"[tiab] OR "glycyrrhiz"[tiab] OR "glycyrrhizin"[tiab] OR "glycyrrhizic acid"[tiab] OR "glycyrrhetinic acid"[tiab] OR "enoxolone"[tiab] OR "carbenoxolone"[tiab] OR "magnesium isoglycyrrhizinate"[tiab] OR "isoglycyrrhizinate"[tiab] OR "glycyrrhizinate"[tiab] OR "diammonium glycyrrhizinate"[tiab] OR "monoammonium glycyrrhizinate"[tiab] OR "ammonium glycyrrhizinate"[tiab] OR "18alpha-glycyrrhetinic acid"[tiab] OR "18beta-glycyrrhetinic acid"[tiab]) AND ("immune checkpoint inhibitor"[tiab] OR "checkpoint inhibitor"[tiab] OR "checkpoint blockade"[tiab] OR "PD-1"[tiab] OR "PD-L1"[tiab] OR "CTLA-4"[tiab] OR "ipilimumab"[tiab] OR "nivolumab"[tiab] OR "pembrolizumab"[tiab] OR "atezolizumab"[tiab] OR "durvalumab"[tiab] OR "cemiplimab"[tiab] OR "avelumab"[tiab] OR "tremelimumab"[tiab] OR "dostarlimab"[tiab] OR "toripalimab"[tiab] OR "sintilimab"[tiab] OR "camrelizumab"[tiab] OR "tislelizumab"[tiab] OR "immune-related adverse event"[tiab] OR "immune-related adverse events"[tiab] OR "immune-related hepatitis"[tiab] OR "checkpoint inhibitor hepatitis"[tiab]) AND ("drug-induced liver injury"[tiab] OR hepatotoxicity[tiab] OR "liver injury"[tiab] OR hepatitis[tiab] OR "immune-related adverse event"[tiab])

S+C+H — Substance AND clinical context AND HMGB1

("Glycyrrhiza"[tiab] OR "licorice"[tiab] OR "liquorice"[tiab] OR "glycyrrhiz"[tiab] OR "glycyrrhizin"[tiab] OR "glycyrrhizic acid"[tiab] OR "glycyrrhetinic acid"[tiab] OR "enoxolone"[tiab] OR "carbenoxolone"[tiab] OR "magnesium isoglycyrrhizinate"[tiab] OR "isoglycyrrhizinate"[tiab] OR "glycyrrhizinate"[tiab] OR "diammonium glycyrrhizinate"[tiab] OR "monoammonium glycyrrhizinate"[tiab] OR "ammonium glycyrrhizinate"[tiab] OR "18alpha-glycyrrhetinic acid"[tiab] OR "18beta-glycyrrhetinic acid"[tiab]) AND ("immune checkpoint inhibitor"[tiab] OR "checkpoint inhibitor"[tiab] OR "checkpoint blockade"[tiab] OR "PD-1"[tiab] OR "PD-L1"[tiab] OR "CTLA-4"[tiab] OR "ipilimumab"[tiab] OR "nivolumab"[tiab] OR "pembrolizumab"[tiab] OR "atezolizumab"[tiab] OR "durvalumab"[tiab] OR "cemiplimab"[tiab] OR "avelumab"[tiab] OR "tremelimumab"[tiab] OR "dostarlimab"[tiab] OR "toripalimab"[tiab] OR "sintilimab"[tiab] OR "camrelizumab"[tiab] OR "tislelizumab"[tiab] OR "immune-related adverse event"[tiab] OR "immune-related adverse events"[tiab] OR "immune-related hepatitis"[tiab] OR "checkpoint inhibitor hepatitis"[tiab]) AND (HMGB1[tiab] OR "high mobility group box 1"[tiab])

Concept blocks as searched

Substance — Glycyrrhiza, licorice, liquorice, glycyrrhiz\, glycyrrhizin, glycyrrhizic acid, glycyrrhetinic acid, 18α-glycyrrhetinic acid, 18β-glycyrrhetinic acid, enoxolone,

carbenoxolone, magnesium isoglycyrrhizinate, isoglycyrrhizinate, monoammonium glycyrrhizinate, diammonium glycyrrhizinate, ammonium glycyrrhizinate.

Clinical context — immune checkpoint inhibitor, checkpoint inhibitor, checkpoint blockade, PD-1, PD-L1, CTLA-4, the individual agents ipilimumab, nivolumab, pembrolizumab, atezolizumab, durvalumab, cemiplimab, avelumab, tremelimumab, dostarlimab, toripalimab, sintilimab, camrelizumab and tislelizumab, and the outcomes immune-related adverse event(s), immune-related adverse events, immune-related hepatitis and checkpoint inhibitor hepatitis.

Mechanism and organ — HMGB1, high mobility group box 1, RAGE, TLR2, TLR4, NLRP3, inflammasome, caspase-1, GSDMD, pyroptosis, drug-induced liver injury, hepatotoxicity, liver injury, hepatoprotection, myocarditis, thyroiditis, hypophysitis, colitis, 11beta-hydroxysteroid dehydrogenase, mineralocorticoid, pseudoaldosteronism, farnesoid X receptor and pregnane X receptor.

Limits of the search

The counts are PubMed/MEDLINE counts. The other five sources were queried with their own syntax and indexing and are not counted, so no number here is a combined figure; a record indexed only outside PubMed/MEDLINE would not appear in them. The searches covered titles and abstracts only, so a study reporting a relevant measurement solely in its full text would be missed. Chinese-language records were reached through the international indexes and, where a record was indexed only in a Chinese database, through that database's record for the item. No per-platform query log and no reproducible hit count are available for that part of the search, so none is reported, and no claim of exhaustive coverage of the Chinese-language literature is made. Records were not screened in duplicate. These limits apply to the negative statements in the main text (Section 2.3).

Section S2. How the evidence is described

No summary grade is applied to the questions in Table 2 or to the steps in Fig. 2. A single scale would rank studies that answer different questions, and the appraisal here is descriptive: each row of Table 2 states the design and the comparator, the sample size, the match between the population and the outcome and the question asked, the precision of the estimate, and the principal sources of bias, together with what the evidence does not support. Regulatory documentation of a product's chemical identity is reported as a product fact rather than as evidence about an effect.

The labels on the steps of Fig. 2 are descriptive in the same way, and each records the strongest evidence identified for that step:

- one delayed-antibody mouse study — a single experiment, in one model, with one agent, reporting a cardiac benefit and no detected short-term tumour-control decrement.
- observational only — human data that are retrospective or uncontrolled.
- not directly tested in the identified literature — no measurement of that step was found among the studies included here.
- not reported — the outcome was not among the reported outcomes of the studies identified, which leaves the question open rather than answered in the negative.

Section S3. Checks of reported quantitative results

S3.1 Cao 2022: AUC values inconsistent with the reported Cmax

Cao et al. (2022) [30] report internally consistent peak concentrations for the mono-glucuronide metabolite and AUC0–T values that are not. Section 3.3 uses only the former.

The AUC units in Tables 8–9 of Cao et al. are inconsistent with those in Tables 6–7: an AUC0–T of 6,625.54 mg·h/L over the 48-h sampling period would imply a mean concentration of about 138 mg/L, roughly 58-fold the reported Cmax of 2.38 mg/L in the same table, and an AUC cannot exceed 48 × 2.38 = 114 mg·h/L if the two columns describe one 0–48 h concentration–time course. Tables 6–7 print ng·h/mL and Tables 8–9 print mg·h/L, so a unit label is one candidate explanation; a thousandfold unit discrepancy would be a different reading of the tables, and the internal inconsistency does not settle which it is, so no value here is corrected or rescaled.

We therefore use the reported Cmax and Tmax for the descriptive comparison in Section 3.3 and do not interpret the AUC values quantitatively. No finding, grade or recommendation in this review depends on them.

S3.2 MAGIC-301: several reported test statistics cannot be reproduced from the published counts

The source reports visit-specific and overall incidence comparisons. Recomputing an uncorrected Pearson chi-square from the aggregate counts the paper itself prints agrees with some of them and disagrees with others:

Comparison (published counts, MgIG versus control)	Reported	Uncorrected Pearson recomputation
Liver biochemical abnormality, days 6–8 (324/765 versus 172/380)	$\chi^2 = 2.4523$, $p = 0.1174$	$\chi^2 = 0.8757$, $p = 0.3494$
Liver biochemical abnormality, days 13–15 (311/765 versus 181/380)	$\chi^2 = 6.3065$, $p = 0.0120$	$\chi^2 = 5.0447$, $p = 0.0247$
Liver biochemical abnormality, overall follow-up (404/765 versus 238/380)	$\chi^2 = 12.6748$, $p = 0.0004$	$\chi^2 = 9.9419$, $p = 0.001616$

Each recomputation uses that time point's published event count and evaluable denominator, without continuity correction. For the overall comparison the direction and the conventional significance judgement agree; for the visit-specific comparisons the discrepancies are larger. Other statistics in the same paper do reproduce from the published counts — rescue hepatoprotective treatment ($\chi^2 \approx 42.07$) and the day 13–15 ALT and AST comparisons ($\chi^2 \approx 16.06$ and 18.55). The source lists χ^2 or Fisher tests in its methods without the further detail that would explain the differences, and a table footnote records one missing data point; applying the full-analysis-set denominator of 766 to the event counts does not reconcile them.

The pattern is therefore partly reproducible and partly not, and it is not attributed here to a single typographical error: a reporting, transcription or analysis difference cannot be distinguished without the underlying data or clarification from the authors. The main text reports the source's own value and states where the recomputation differs (Section 7.1 and Table 1); no value is corrected or rescaled.

Section S4. Supporting detail: preclinical liver-injury models and the proposed trial design

S4.1 T-cell-mediated liver-injury models

In concanavalin A (ConA) hepatitis, the model most often used to represent T-cell-mediated liver injury, protection with MgIG has been attributed to inhibition of autophagy [46], to modulation of p38 and JNK MAPK signalling with parallel effects on inflammatory macrophages [19], and to inhibition of proliferation of the $CD4^{+}CD25^{-}CD69^{+}$ subset [44]; the last is also the clearest English-language statement of the product's configuration, identifying MgIG as "a magnesium salt of 18-α GA stereoisomer" (Section 3.3).

Other members of the class have been tested in the same model: glycyrrhizic acid acted on monocyte-derived macrophages, moving them away from a pro-inflammatory $iNOS^{hi}$ subset and towards an anti-inflammatory $F4/80^{fio}CD11b^{hi}MHCII^{hi}$ one [98]; glycyrrhizin regulated the $CD4^{+}$ T-cell response during fibrogenesis through JNK, ERK and PI3K/AKT signalling [99]; and diammonium glycyrrhizinate acted by inhibiting natural killer T-cell proliferation [100]. Earlier reports describe consistent protection with reduced oxidative and inflammatory markers in the same model [18] and in a 13-week D-galactosamine model reported only at abstract level [16] (Table S2). The class has also been tested outside the organ that was injured: in a mouse ConA hepatitis model, glycyrrhizin attenuated renal inflammation through the IL-25/M2 axis [101].

S4.2 Proposed trial design (sketch)

- Population. Adults with solid tumours receiving ICIs who develop ChILI for which corticosteroid treatment is clinically indicated. A treatment design is more conservative than prophylaxis, which commits patients to treatment before injury.
- Intervention. Isomer-specified MgIG at the published dose, 200 mg/day [1], with a dose-finding run-in.
- Comparator. Both arms receive corticosteroid treatment, which is clinically indicated here; the randomised question is whether MgIG is added to it, as MgIG plus standard corticosteroid against matched placebo plus standard corticosteroid.
- Sample size. See Section 12 of the main text for the calculation: with 1:1 allocation, a two-sided alpha of 0.05 and 80% power, detecting an increase in day-10 ALT normalisation from 17.4% with corticosteroid alone [1] to 25% requires about 451 evaluable patients per arm (497 per arm allowing for 10% dropout; 994 in total). Powering for the full observed difference would roughly halve that requirement.
- Blinding and standardisation. A placebo-controlled, double-blind adjunctive design is preferable where feasible, since the intervention is an infusion and a matched placebo is straightforward to prepare; otherwise open-label treatment with blinded endpoint adjudication is acceptable. Isomer identity and purity must be prespecified as product quality attributes.

S4.3 The ferroptosis-plus-HMGB1-blockade platform in detail

The platform's carrier releases glycyrrhizic acid in the tumour's reductive environment, and the authors compared vehicle, the iron carrier alone, the carrier without glycyrrhizic acid, and the loaded platform, so the increment attributable to the compound is measured rather than inferred [86]. The compound-free platform did not substantially inhibit tumour growth; the loaded platform did, with half the mice alive beyond 70 days, reduced granulocytic MDSCs and raised $CD8^{+}$ T-cell IFN-γ expression, whereas ferroptosis alone increased granulocytic MDSC infiltration and their suppressive activity. Two things the model does not establish: it does not isolate HMGB1 as the causal step, because no HMGB1-neutralising or HMGB1-deficient control was used and the $CD8^{+}$ T-cell increase appeared with the compound-free platform as well; and it does not test release followed by blockade, since the compound is delivered with the ferroptosis

inducer rather than after it. In the same model the combination with anti-PD-L1 suppressed tumour growth further, and the survival curve of that group lies above the controls, with the treated groups declining to about half surviving over the follow-up shown; no statistical test of that difference is verified in this review. The platform has been demonstrated only in the 4T1 model, in which irAE and antitumour outcomes were not assessed together, and no patient data exist.

Notes on supplementary content

Table S1 expands the "Zhi 2025" row of Table 1 in the main text. Table S2 is a working table of compound-specific preclinical studies rather than an exhaustive census: it tabulates 39 entries across six panels, and sources it omits — including several of the adjacent constituents of Section 5.2 and most of the delivery designs of Section 6 — are cited in the text. Sections S1 to S3 carry the search strategy, the way the evidence is described in Table 2 and Fig. 2, and checks of reported quantitative results (Cao 2022 in S3.1; MAGIC-301 in S3.2); Section S4 collects the preclinical model detail of Section 7.2, the design sketch behind the trial proposed in Section 12, and the platform analysis of Section 6.3.
